# Supplementary figures and images for: Mutation bias interacts with composition bias to influence adaptive evolution
Source: PLoS Comput Biol. 2020 Sep 28;16(9):e1008296. doi: 10.1371/journal.pcbi.1008296 (PMC7571706; doi:10.1371/journal.pcbi.1008296)

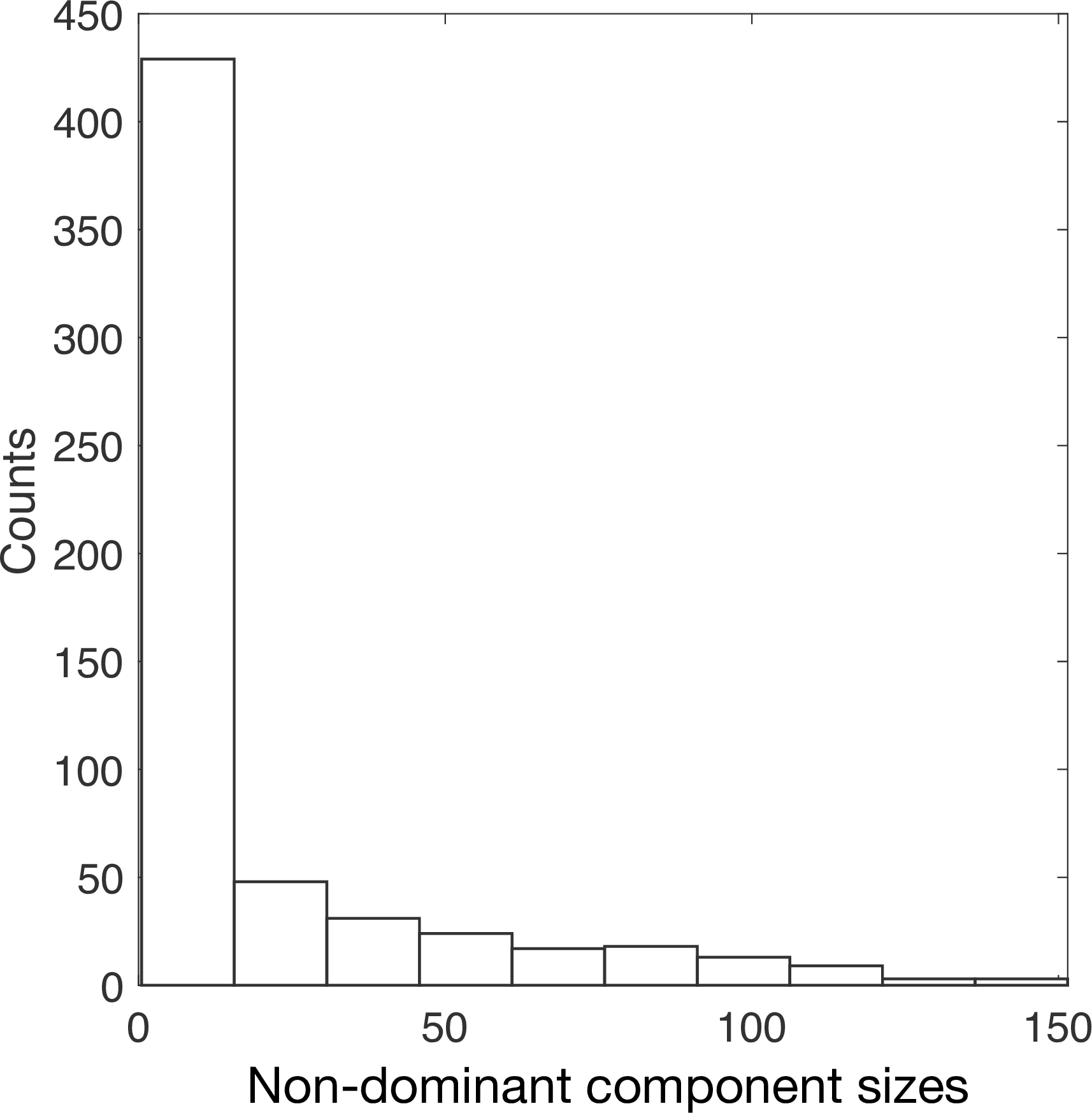

Supplement: S1 Fig — Histogram of non-dominant component sizes. In total, 46% are singletons and 97% are not large enough to satisfy our inclusion criterion of containing 100 sequences. (TIF) [file pcbi.1008296.s001.tif]

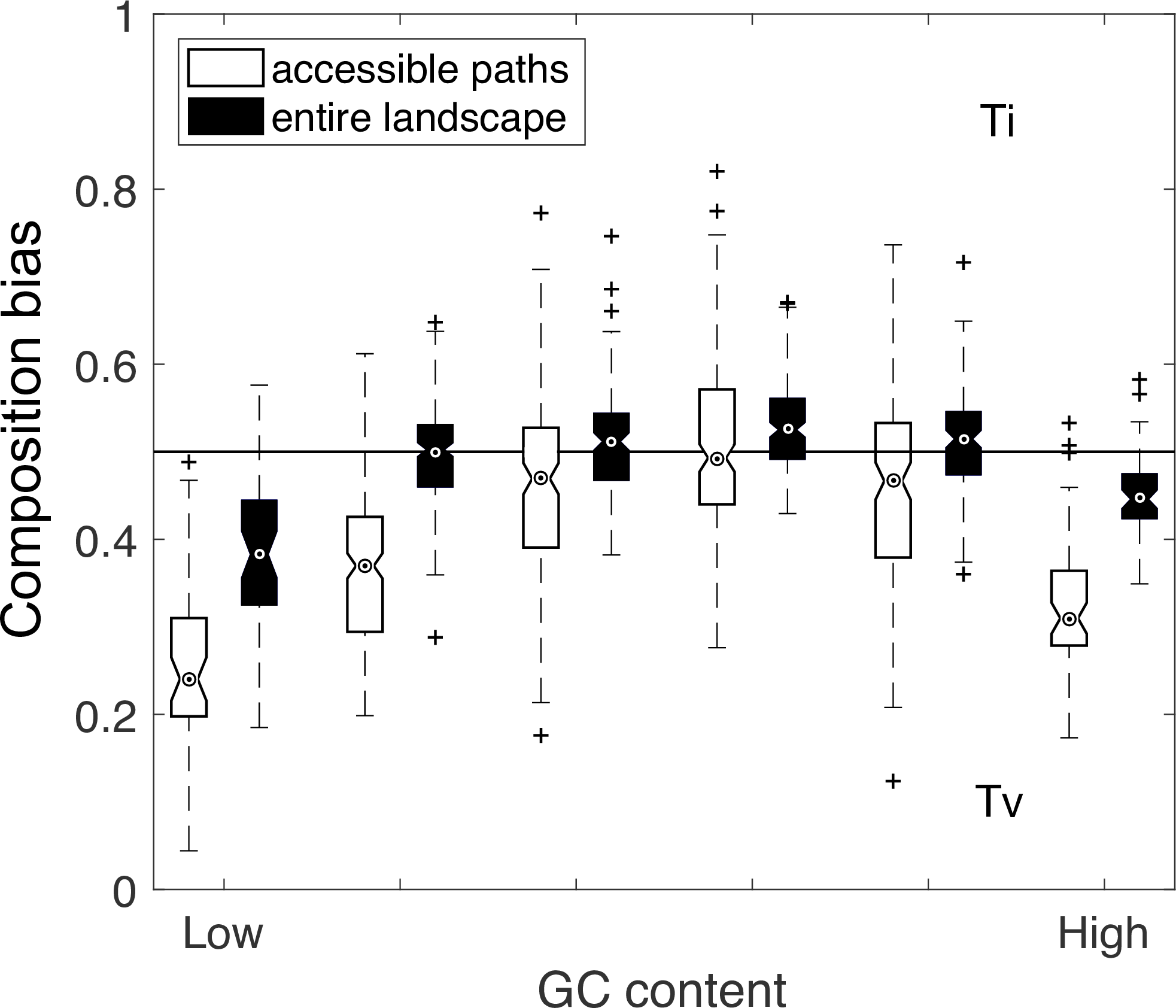

Supplement: S2 Fig — The composition bias in entire landscapes, and in accessible mutational paths connecting the 10% of binding sites with the lowest affinity to the global peak, is shown in relation to the average GC content of the sequences in the landscape. Data pertain to all 746 landscapes. Notches indicate medians, whiskers indicate the 25th and 75th percentiles, and cross symbols indicate outliers. The horizontal line indicates no composition bias (0.5). (TIF) [file pcbi.1008296.s002.tif]

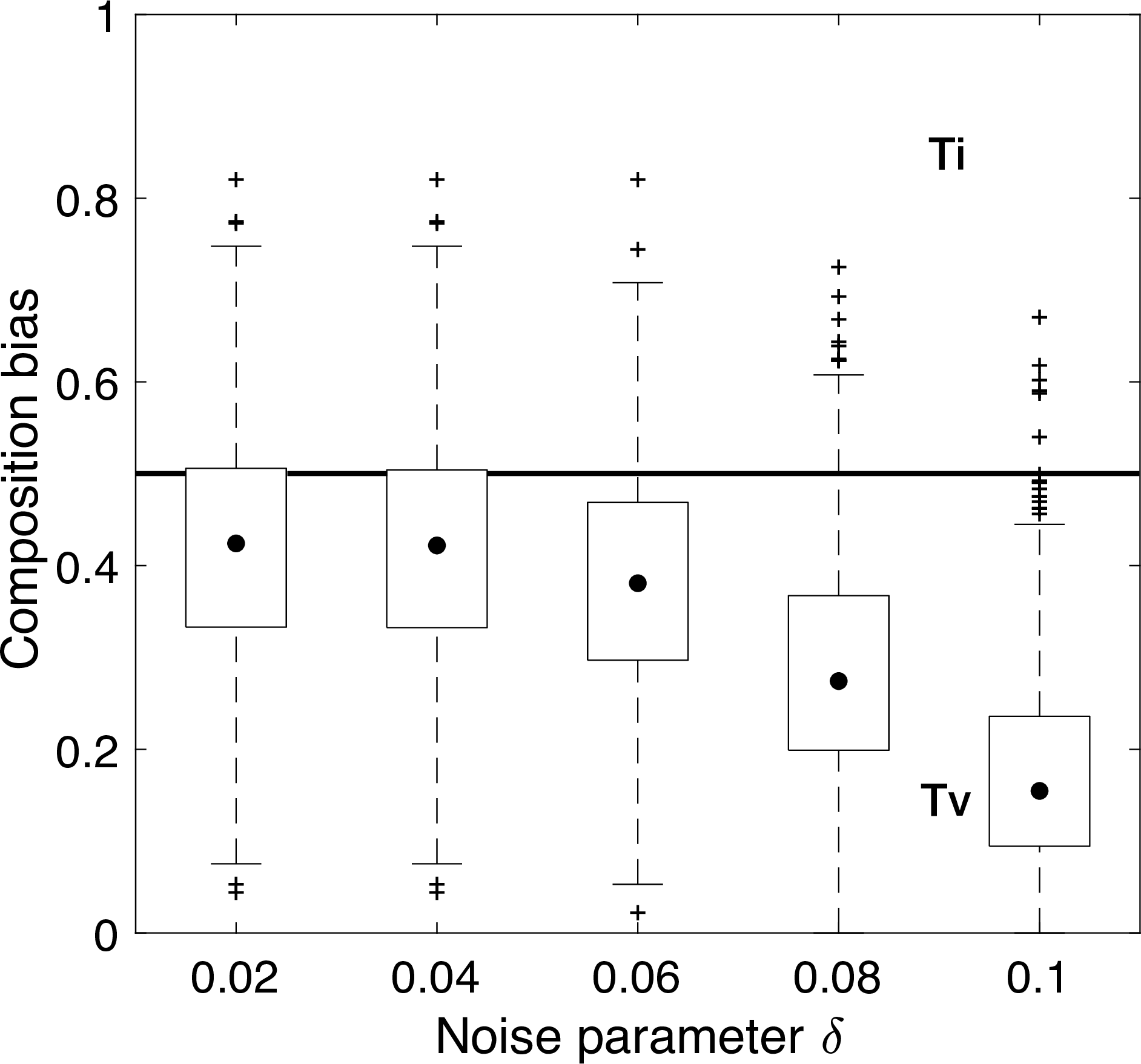

Supplement: S3 Fig — For each landscape and for five different values of the noise threshold δ, we calculated the composition bias along the accessible mutational paths connecting the 10% of binding sites with the lowest affinity to the global peak. Data pertain to all 746 landscapes. Black dots indicate medians, whiskers indicate the 25th and 75th percentiles, and cross symbols indicate outliers. (TIF) [file pcbi.1008296.s003.tif]

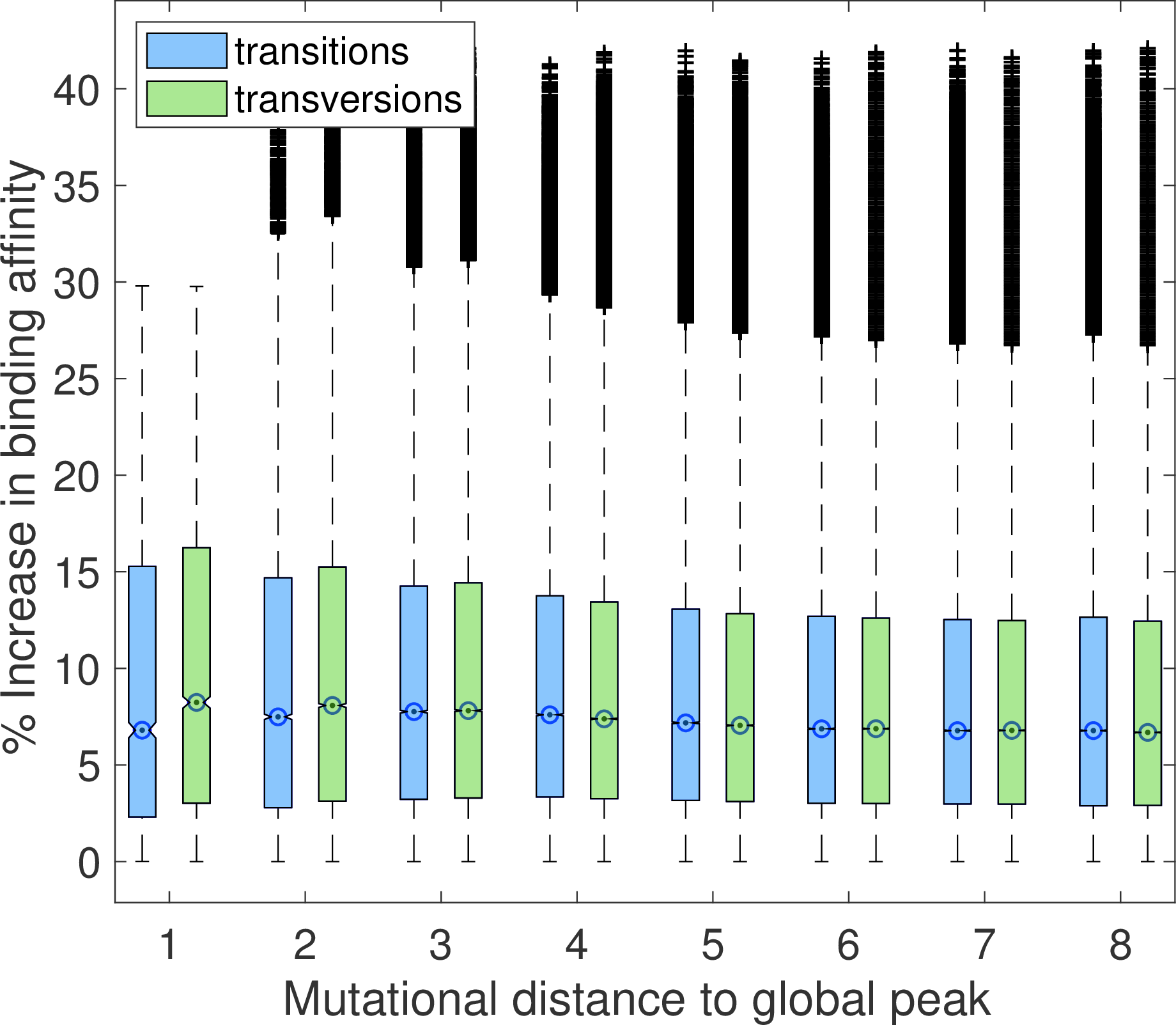

Supplement: S4 Fig — The % increase in binding affinity conferred by transition and transversion mutations along accessible mutational paths is shown in relation to the mutational distance of a binding site to the global peak. For each binding site in each accessible path at each mutational distance d, we calculated the increase in affinity as the percentage change at mutational distance d − 1 along the path, relative to the affinity of the binding site at distance d. Notches indicate medians, whiskers indicate the 25th and 75th percentiles, and cross symbols indicate outliers. Mutational distances 1 and 2 exhibit statistically significant differences in the increase in binding affinity conferred by transitions and transversions (Bonferroni corrected two-sample t test, q < 10−3 and q < 0.05, respectively). (TIF) [file pcbi.1008296.s004.tif]

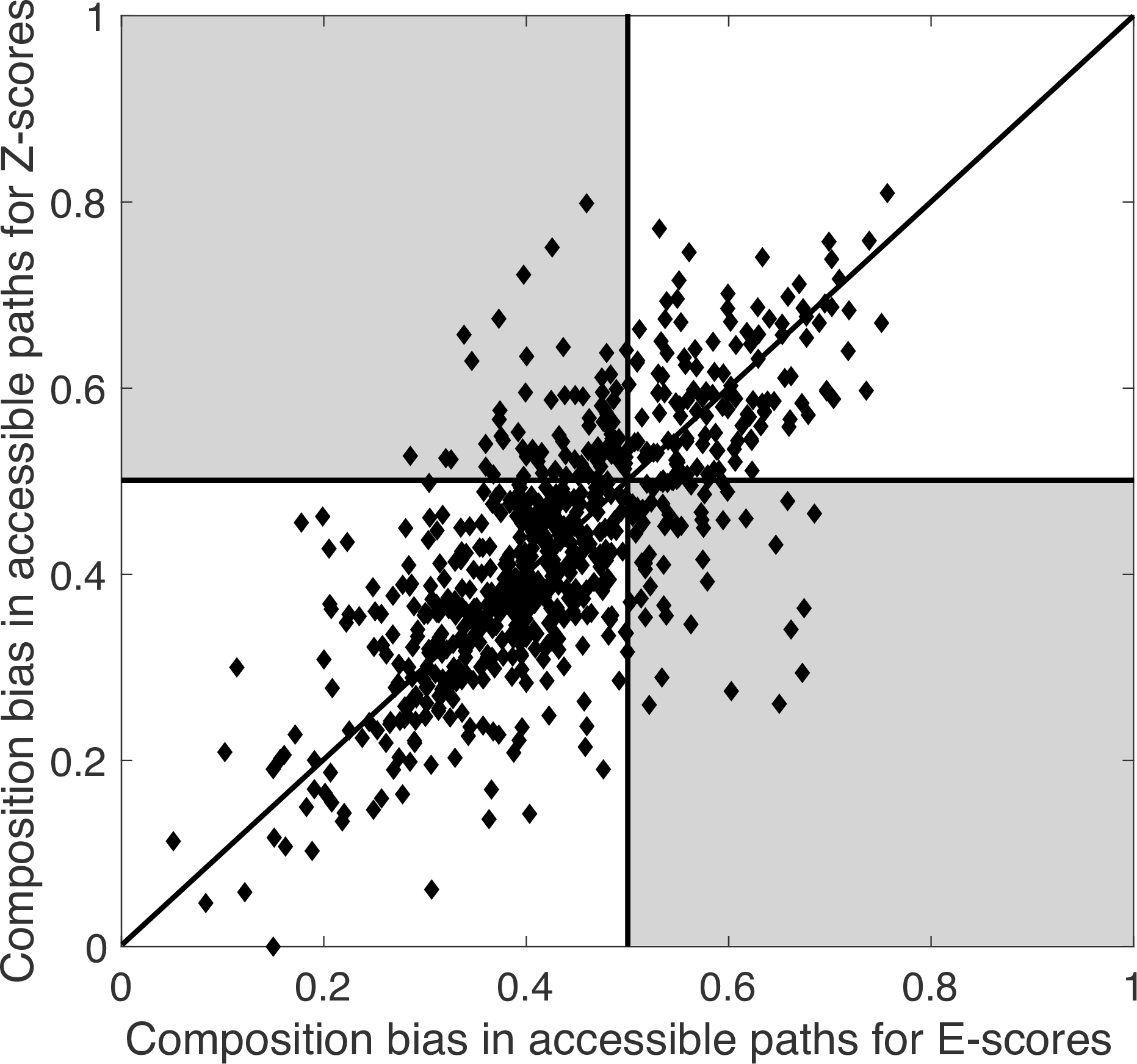

Supplement: S5 Fig — Pearson’s correlation coefficient r = 0.7212, p < 10−10. Data pertain to all 746 landscapes. The shaded gray regions highlight the 83 landscapes that switch from exhibiting a composition bias toward transversions to a composition bias toward transitions (or vice versa). (TIF) [file pcbi.1008296.s005.tif]

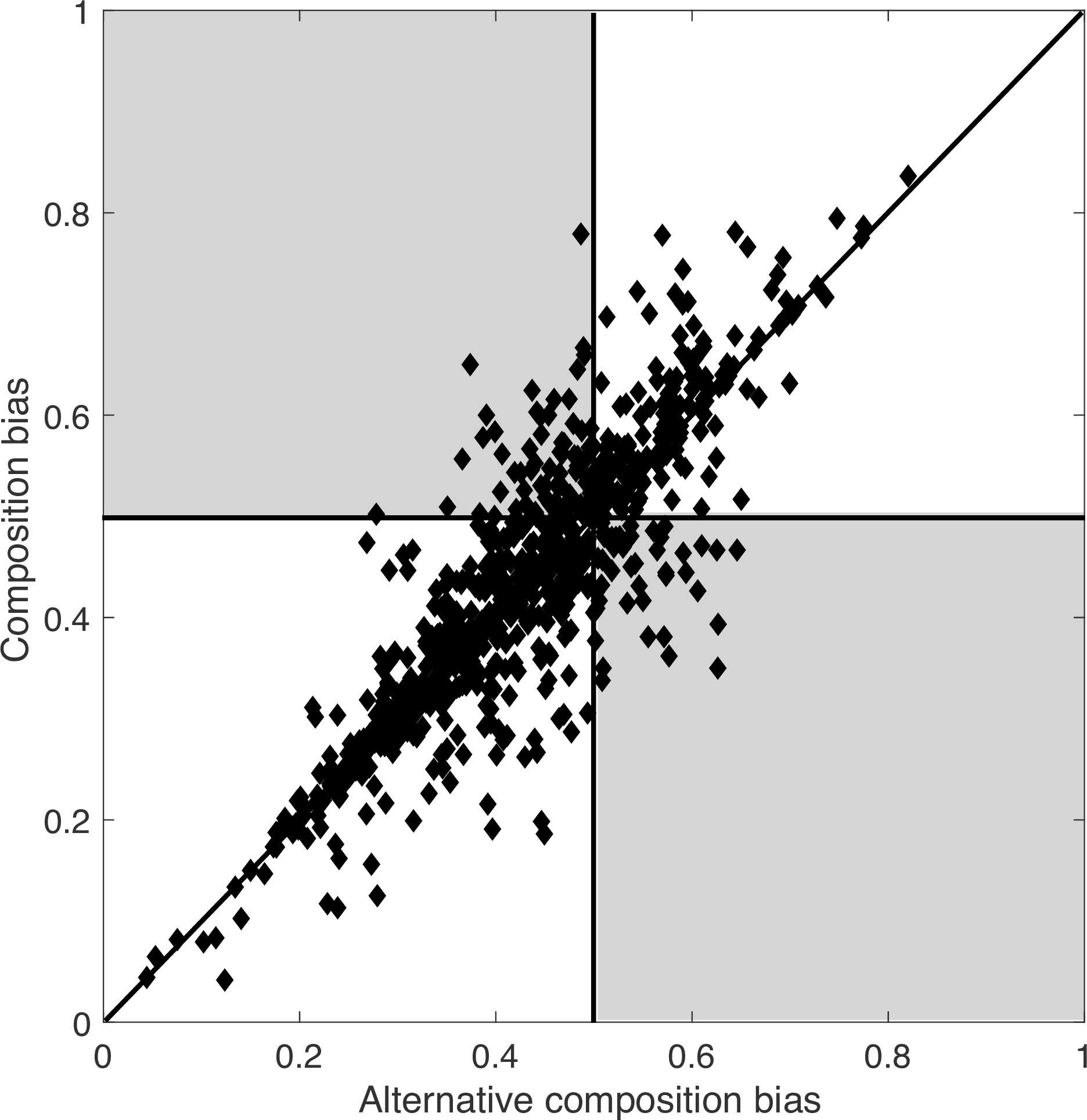

Supplement: S6 Fig — Data pertain to all 746 landscapes, Pearson’s correlation coefficient(r = 0.8541, p < 10−12). The shaded gray regions highlight the 77 landscapes that switch from exhibiting a composition bias toward transversions to a composition bias toward transitions (or vice versa). (TIF) [file pcbi.1008296.s006.tif]

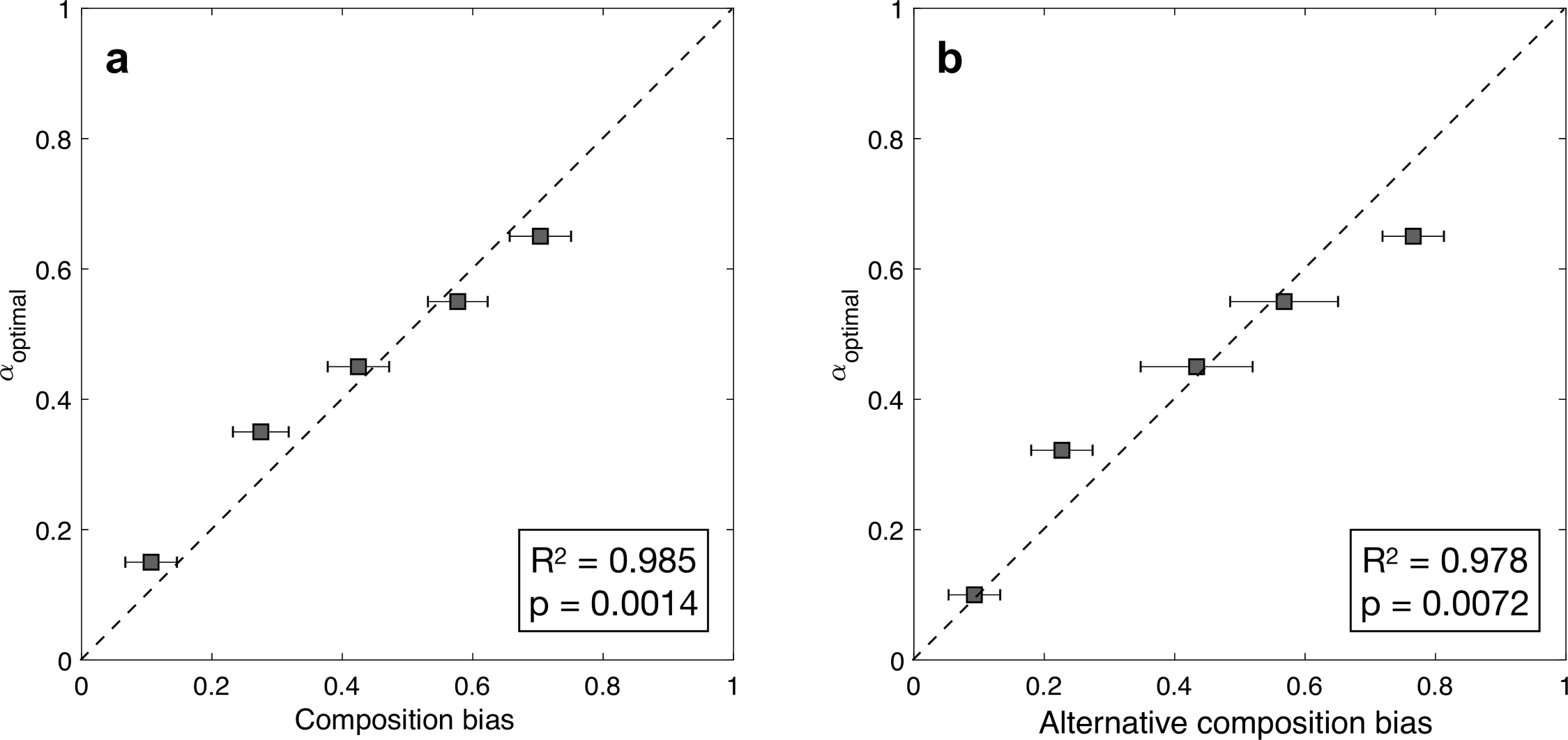

Supplement: S7 Fig — In (a), each step in an accessible mutational path increases binding affinity by at least δ. In (b), each step in an accessible mutational path does not decrease binding affinity more than δ. Data pertain to all 746 landscapes, each of which has its own noise threshold δ. (TIF) [file pcbi.1008296.s007.tif]

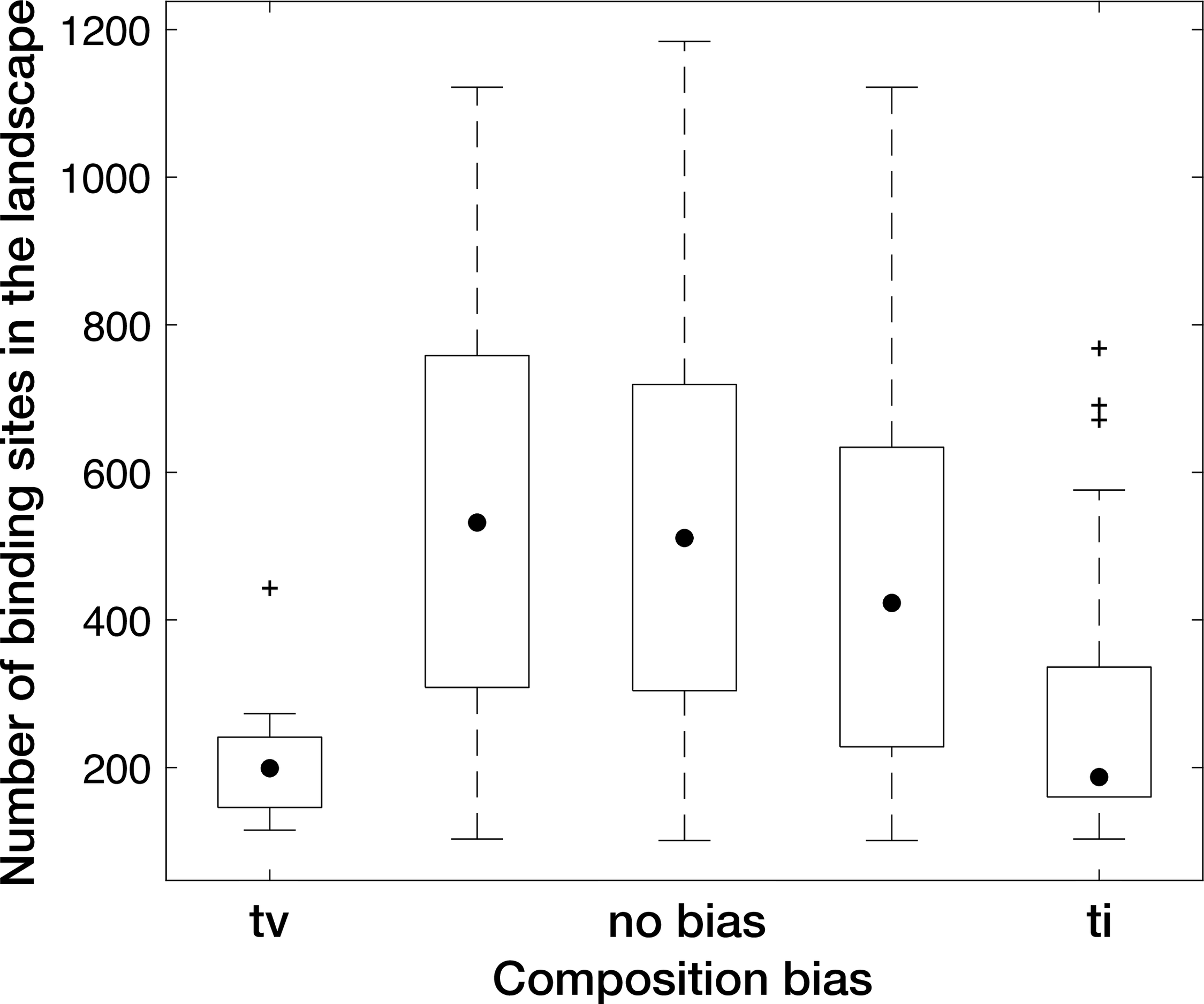

Supplement: S8 Fig — The number of binding sites per landscape is shown in relation to composition bias. Landscapes are grouped as in Fig 3. Data pertain to all 746 landscapes. Black dots indicate medians, whiskers indicate the 25th and 75th percentiles, and cross symbols indicate outliers. (TIF) [file pcbi.1008296.s008.tif]

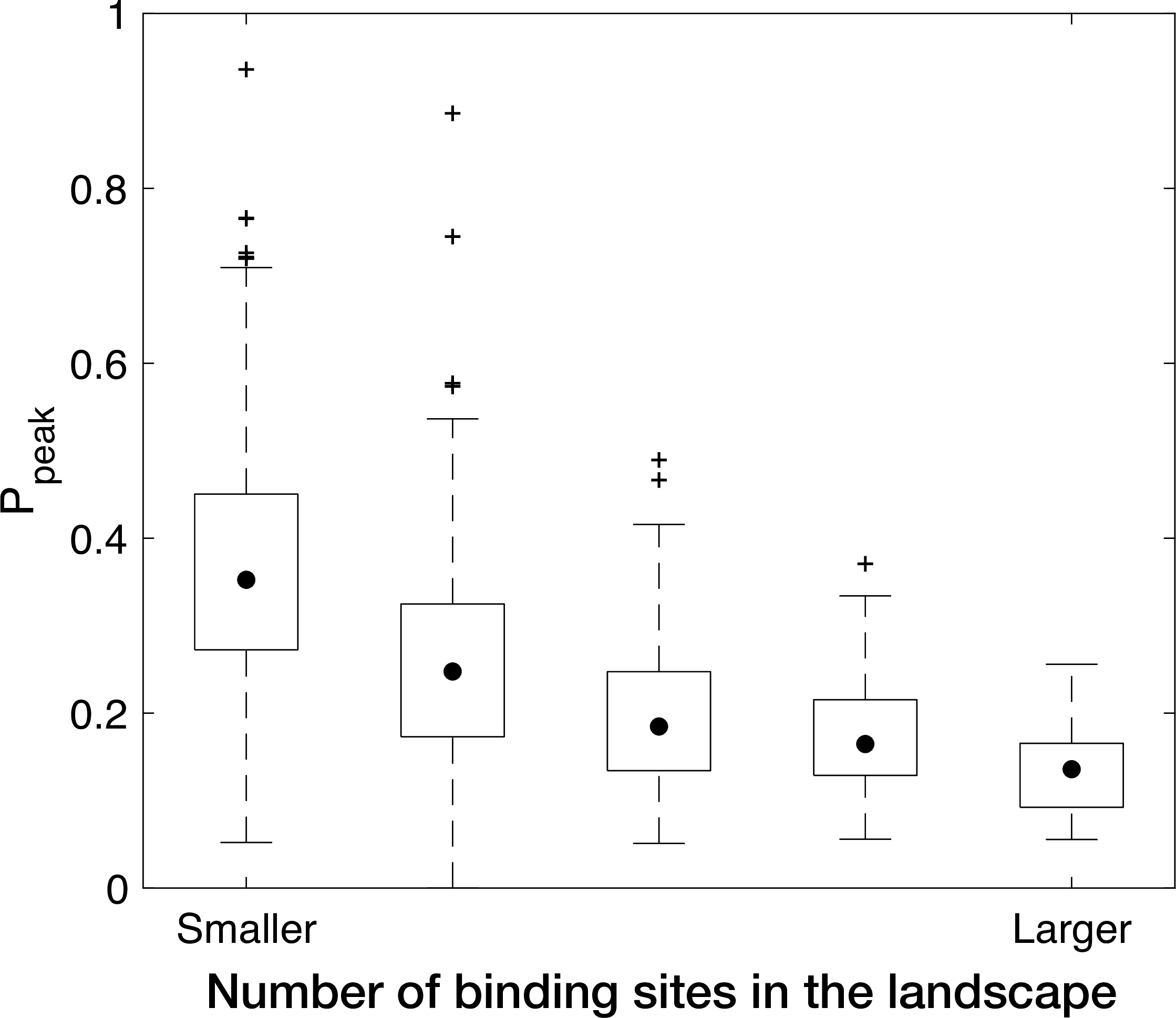

Supplement: S9 Fig — Landscapes are grouped based on the number of binding sites they comprise, with the average number of binding sites per landscape per group ranging from 165.07 to 1037.60 in five linearly spaced increments. The probability of evolving to the global peak in the absence of mutation bias (α = 0.5) is shown in relation to landscape size. Data pertain to all 746 landscapes. Black dots indicate medians, whiskers indicate the 25th and 75th percentiles, and cross symbols indicate outliers. (TIF) [file pcbi.1008296.s009.tif]

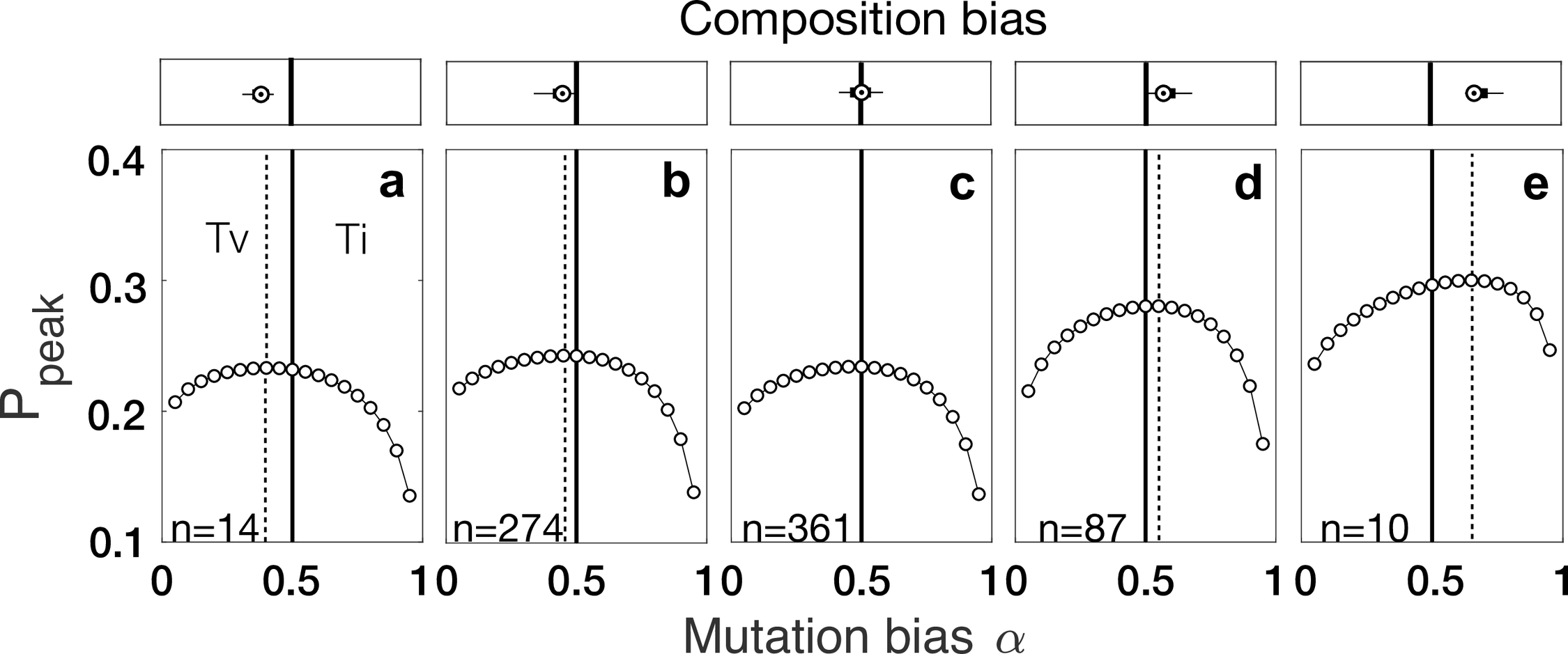

Supplement: S10 Fig — The probability Ppeak of reaching the global peak is shown for 19 different values of the mutation bias parameter α. The solid vertical lines indicate no bias in mutation supply (α = 0.5) and the dashed vertical lines indicate the value of α that maximizes Ppeak. Landscapes are grouped based on their composition bias and the distribution of composition bias per panel is shown on top of each panel. The number of landscapes per panel is indicated is the bottom left corner. Fitness is a function of binding affinity (E-score) using the Gaussian function exp(−((E − Eopt)/σ)2), where E is the E-score of a binding site, Eopt is the optimal E-score, and σ is the variance parameter. Here, Eopt = 0.35 (the lowest E-score in our landscapes) and σ = 0.1. (TIF) [file pcbi.1008296.s010.tif]

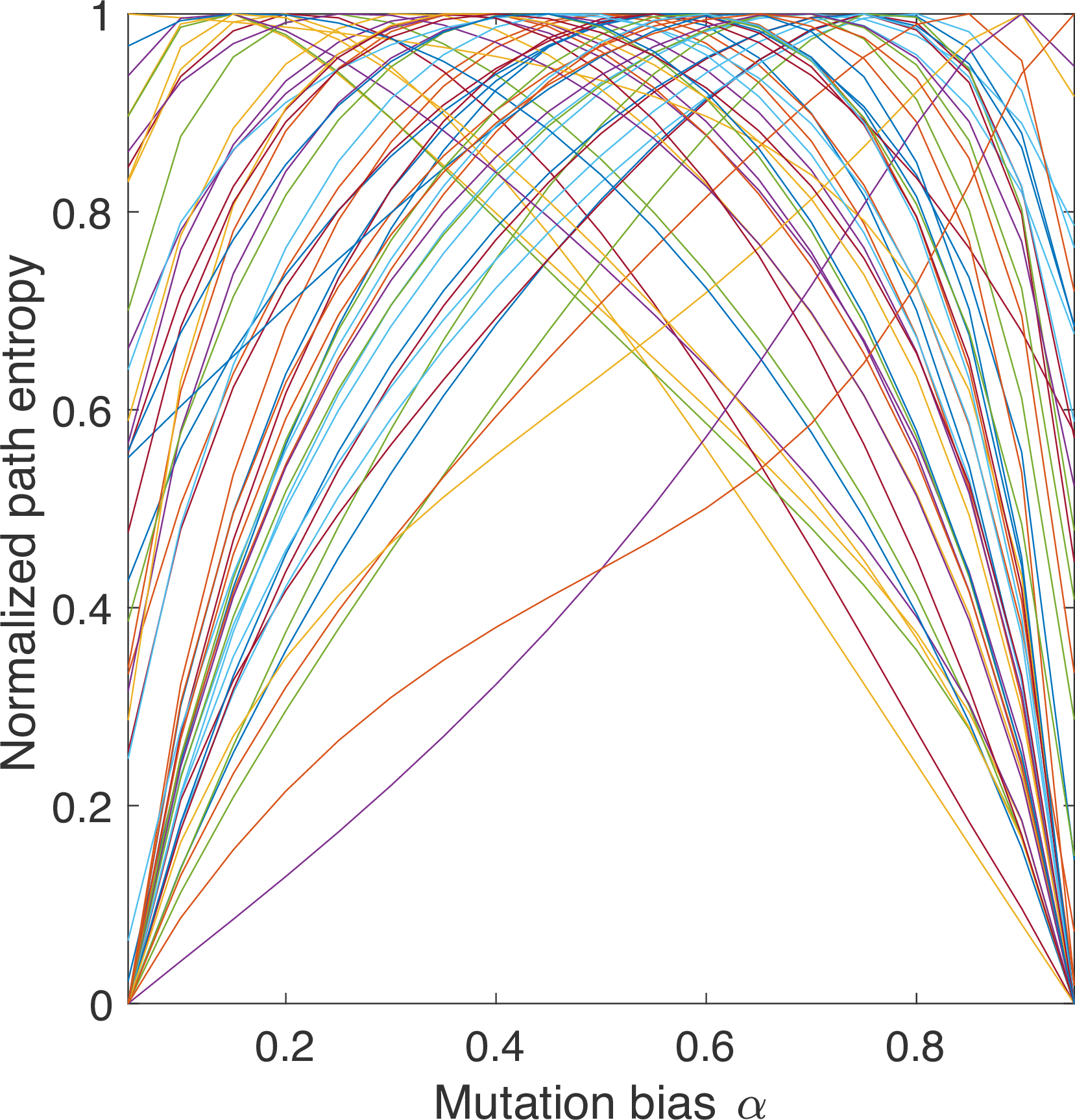

Supplement: S11 Fig — Data pertain to 50 randomly chosen landscapes for 19 different values of the mutation bias parameter α. (TIF) [file pcbi.1008296.s011.tif]

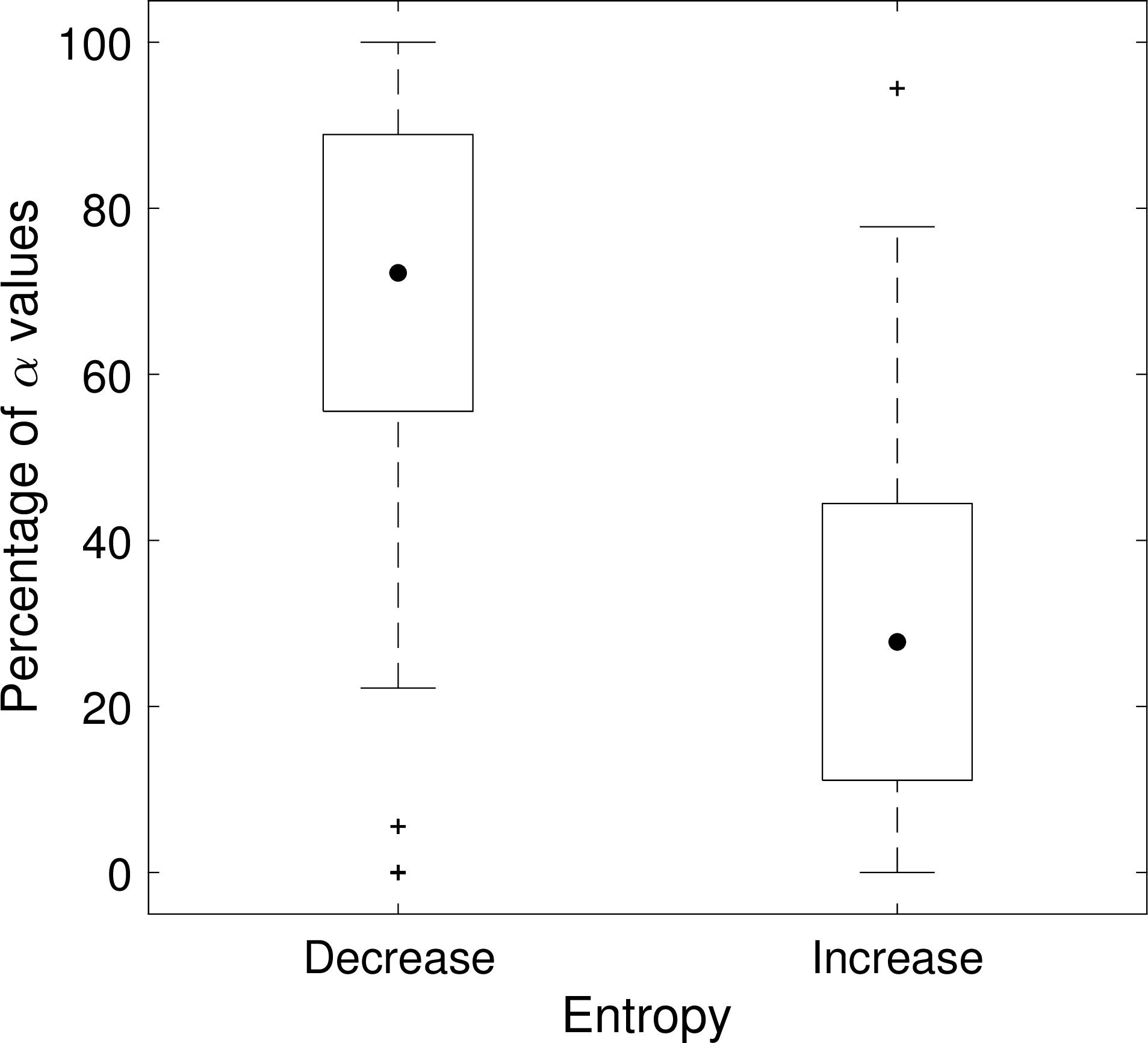

Supplement: S12 Fig — Shown are the percentage of mutation bias values α that increase or decrease path entropy (which is inversely related to the predictability of evolution), relative to when there is no mutation bias (α = 0.5). Data pertain to all 19 values of the mutation bias parameter α on each of the 746 landscapes. Black dots indicate medians, whiskers indicate the 25th and 75th percentiles, and cross symbols indicate outliers. (TIF) [file pcbi.1008296.s012.tif]

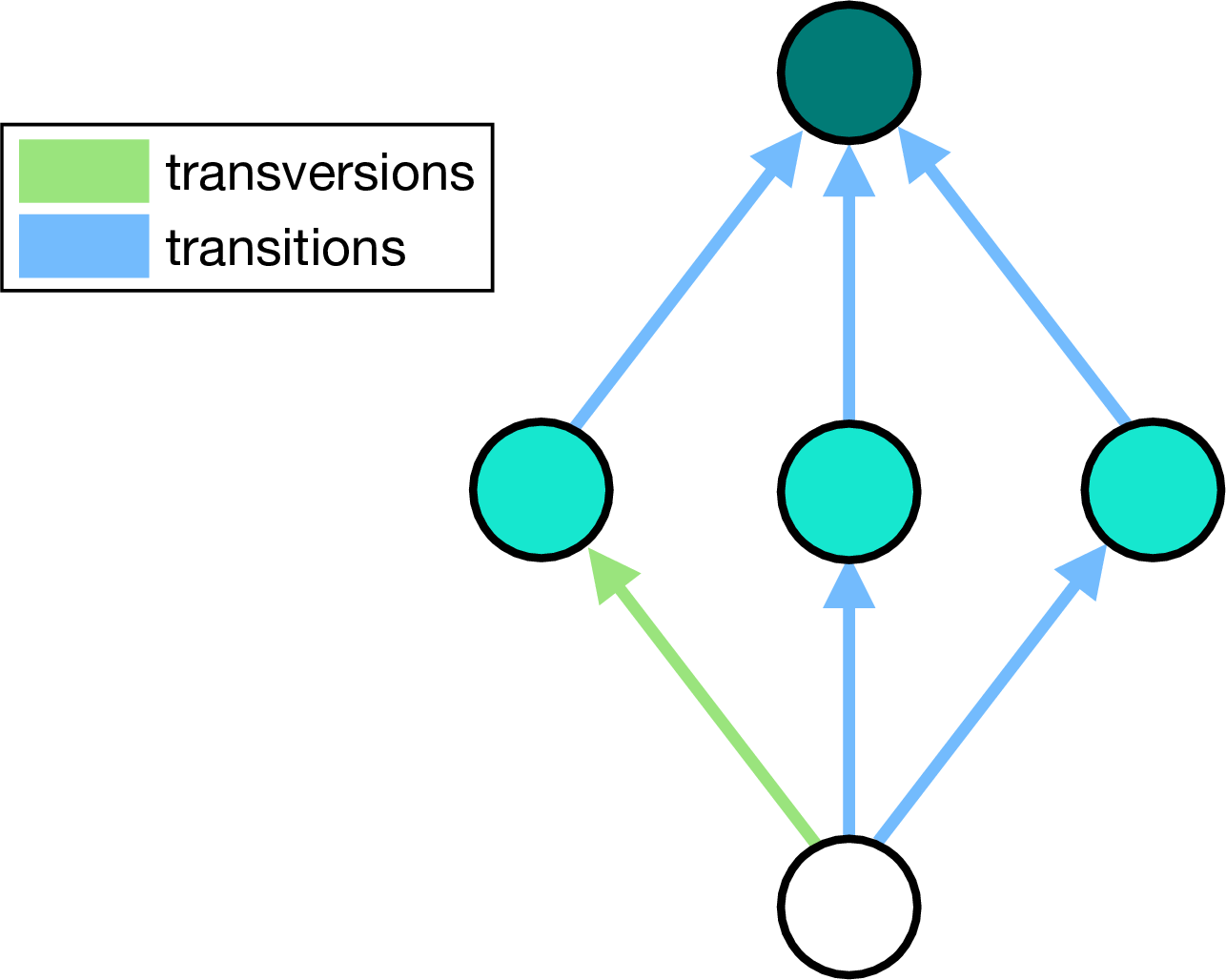

Supplement: S13 Fig — Nodes represent sequences in a landscape, and directed edges represent accessible mutations between sequences. Edge colors represent mutation type and node colors represent binding affinity (darker = higher). This landscape exhibits a strong composition bias toward transitions. Path entropy is therefore minimized by a strong mutation bias toward transversions, because an evolving population will utilize only one of the three accessible mutational paths. Conversely, one might expect path entropy to be maximized by a strong mutation bias toward transitions. However, this is not the case, because an evolving population will only utilize two of the three accessible mutational paths. The mutation bias that maximizes path entropy is actually the one that makes the three first-step mutations equiprobable. In more complex scenarios, with more and longer paths that include a greater diversity of binding affinities and more heterogeneous distributions of mutation types, a single summary statistic like composition bias is unlikely to accurately predict the value of mutation bias that maximizes path entropy. (TIF) [file pcbi.1008296.s013.tif]

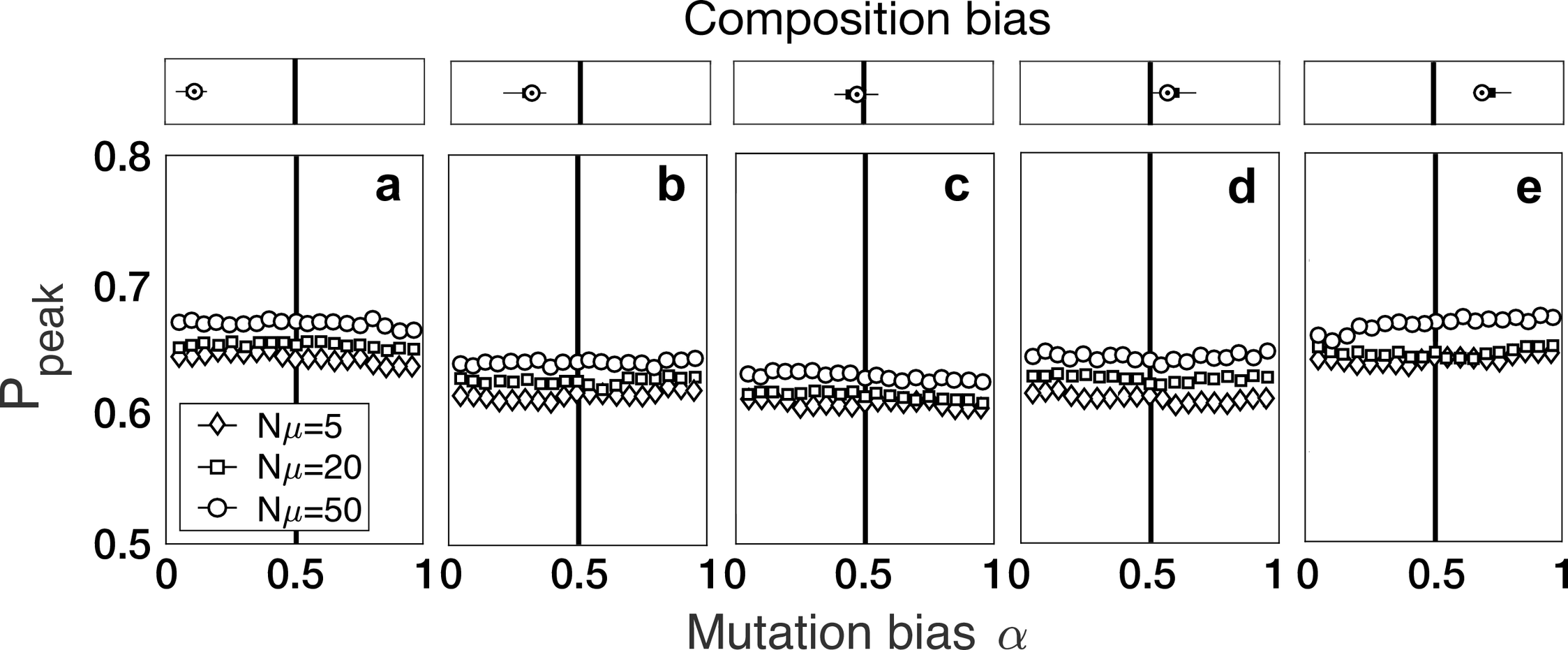

Supplement: S14 Fig — The probability Ppeak of reaching the global peak is shown for 19 different values of the mutation bias parameter α. This probability is calculated as the proportion of simulations in which at least half of the population evolves to the global peak. The solid vertical lines indicate no bias in mutation supply (α = 0.5). Landscapes are grouped based on their composition bias and the distribution of composition bias per panel is shown on top of each panel as in Fig 3. (TIF) [file pcbi.1008296.s014.tif]

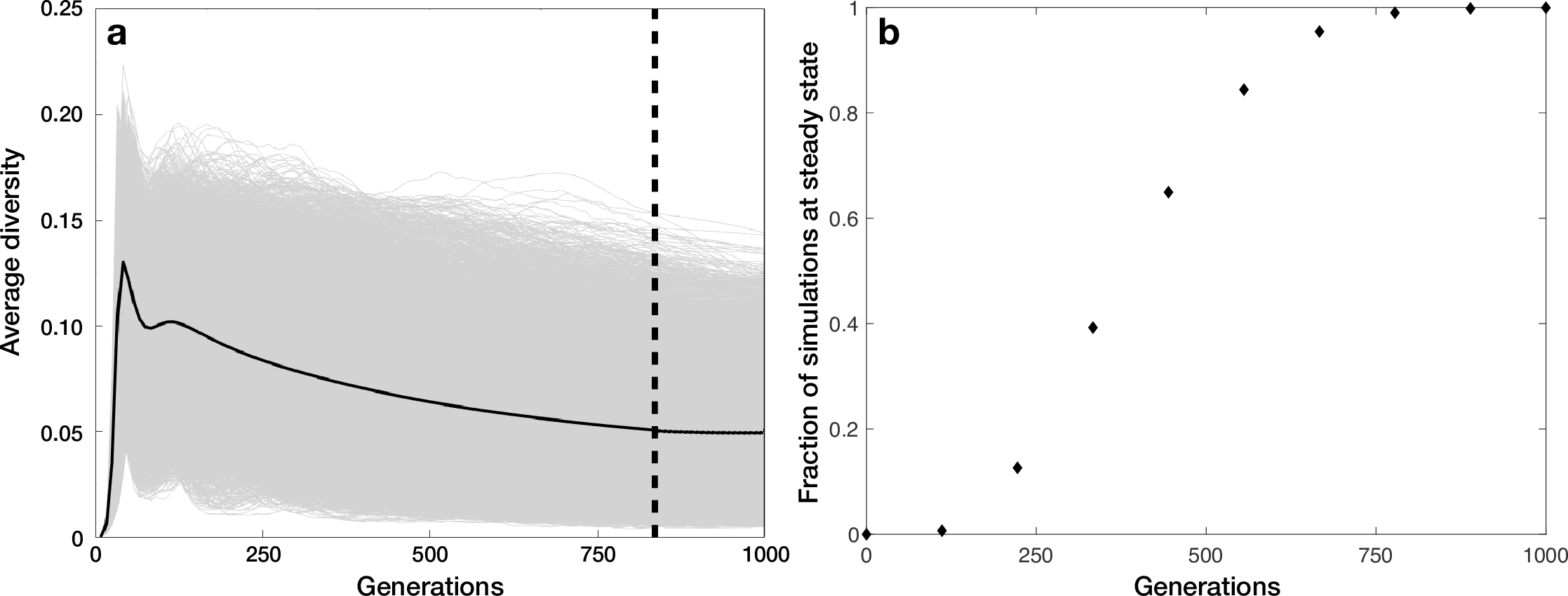

Supplement: S15 Fig — (a) The average Shannon’s diversity is shown in relation to the number of generations. After 827 generations, more than 99.9% of the 4, 252, 200 simulations reached steady state diversity levels within a tolerance of 0.01% of the final diversity level. The black line shows the average across all simulations. (b) The fraction of simulations that have reached steady state diversity levels is shown in relation to generation number. (TIF) [file pcbi.1008296.s015.tif]

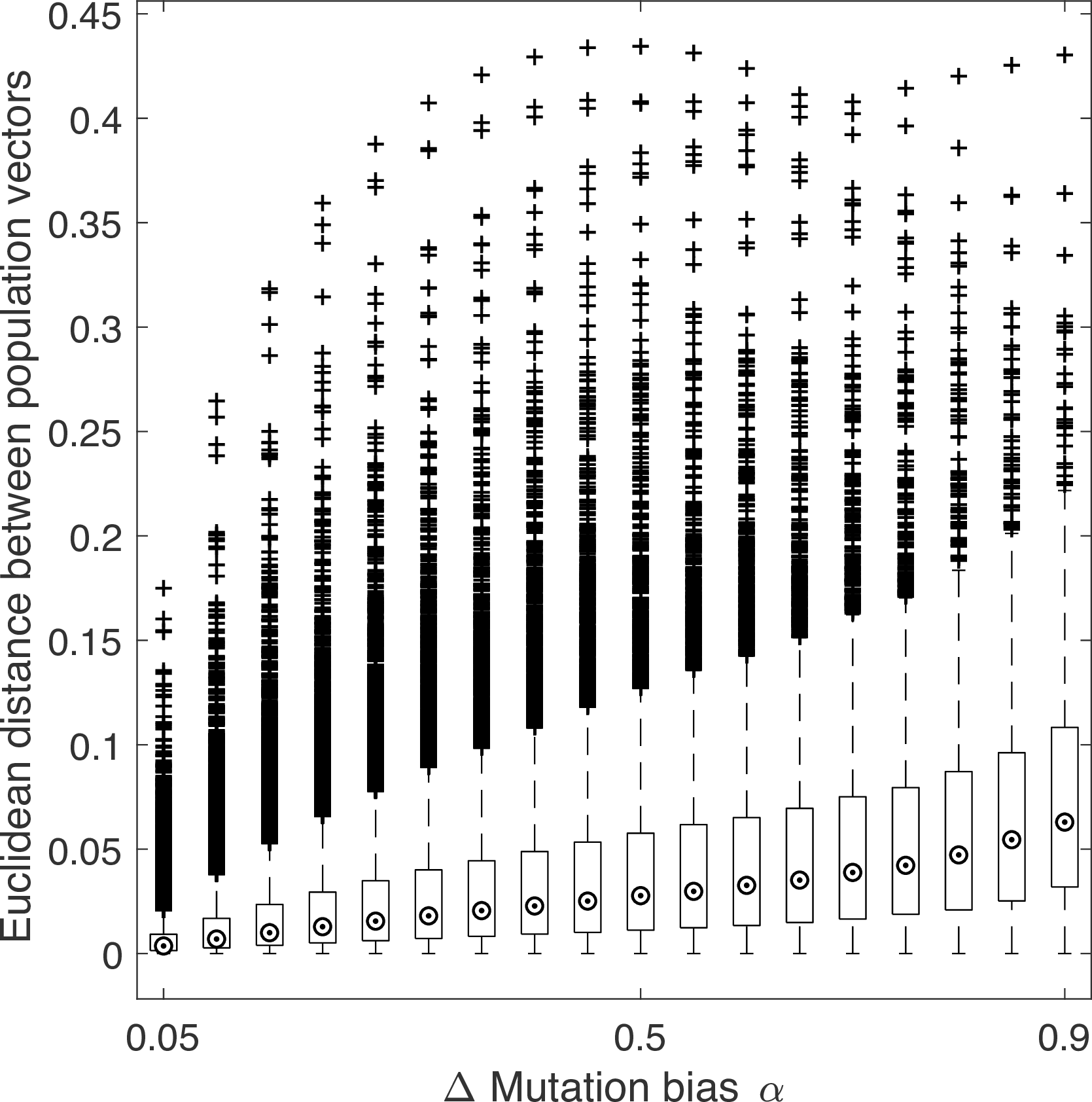

Supplement: S16 Fig — We characterized the steady state distribution of infinite populations as the eigenvector that corresponds to the largest eigenvalue of the matrix P (Methods). For any pair of such populations, we measured their overlap as the Euclidean distance between these eigenvectors—the shorter the distance, the higher the overlap. This panel shows this distance for pairs of populations in relation to the difference in their mutation bias parameters. (TIF) [file pcbi.1008296.s016.tif]

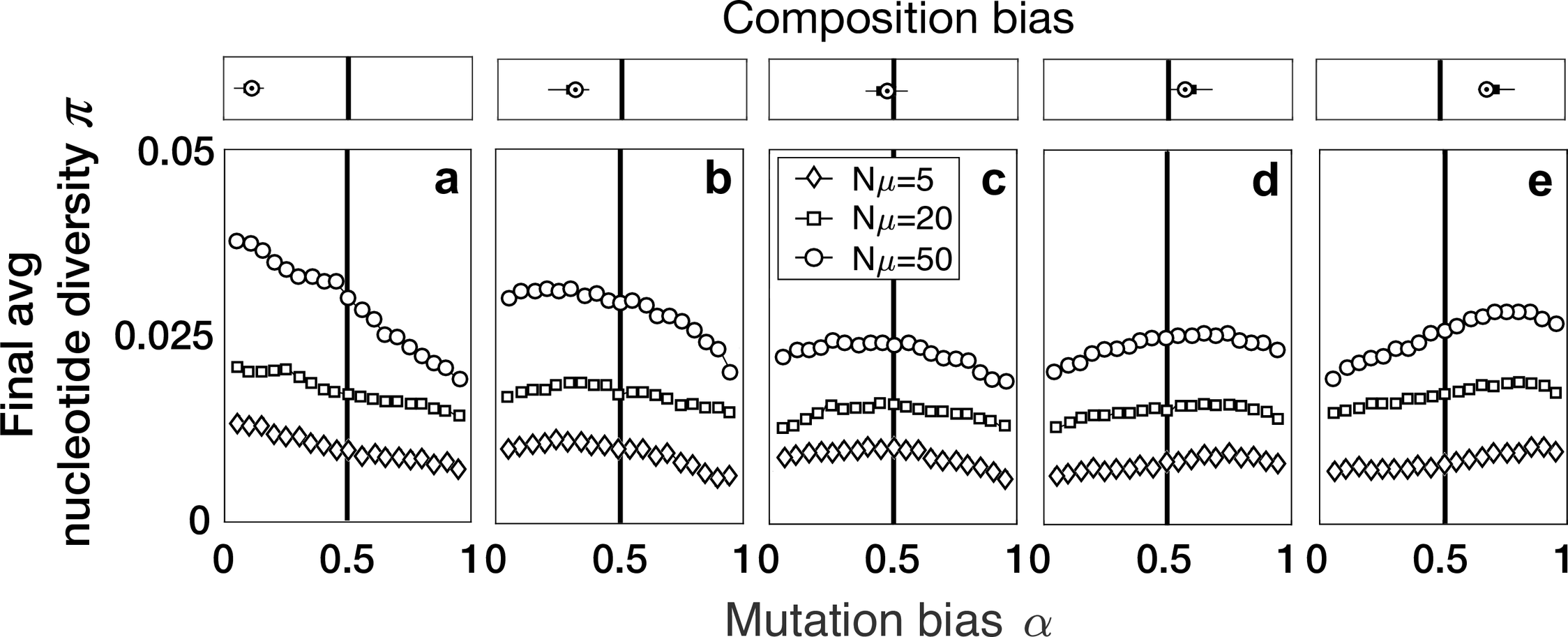

Supplement: S17 Fig — (a-e) The final average nucleotide diversity of evolved populations at steady state is shown for 19 different values of the mutation bias parameter α, and for each of three different values of mutation supply Nμ (see legend). The solid vertical lines indicate no bias in mutation supply (α = 0.5). Landscapes are grouped based on their composition bias and the distribution of composition bias per panel is shown on top of each panel, as in Fig 3. (TIF) [file pcbi.1008296.s017.tif]

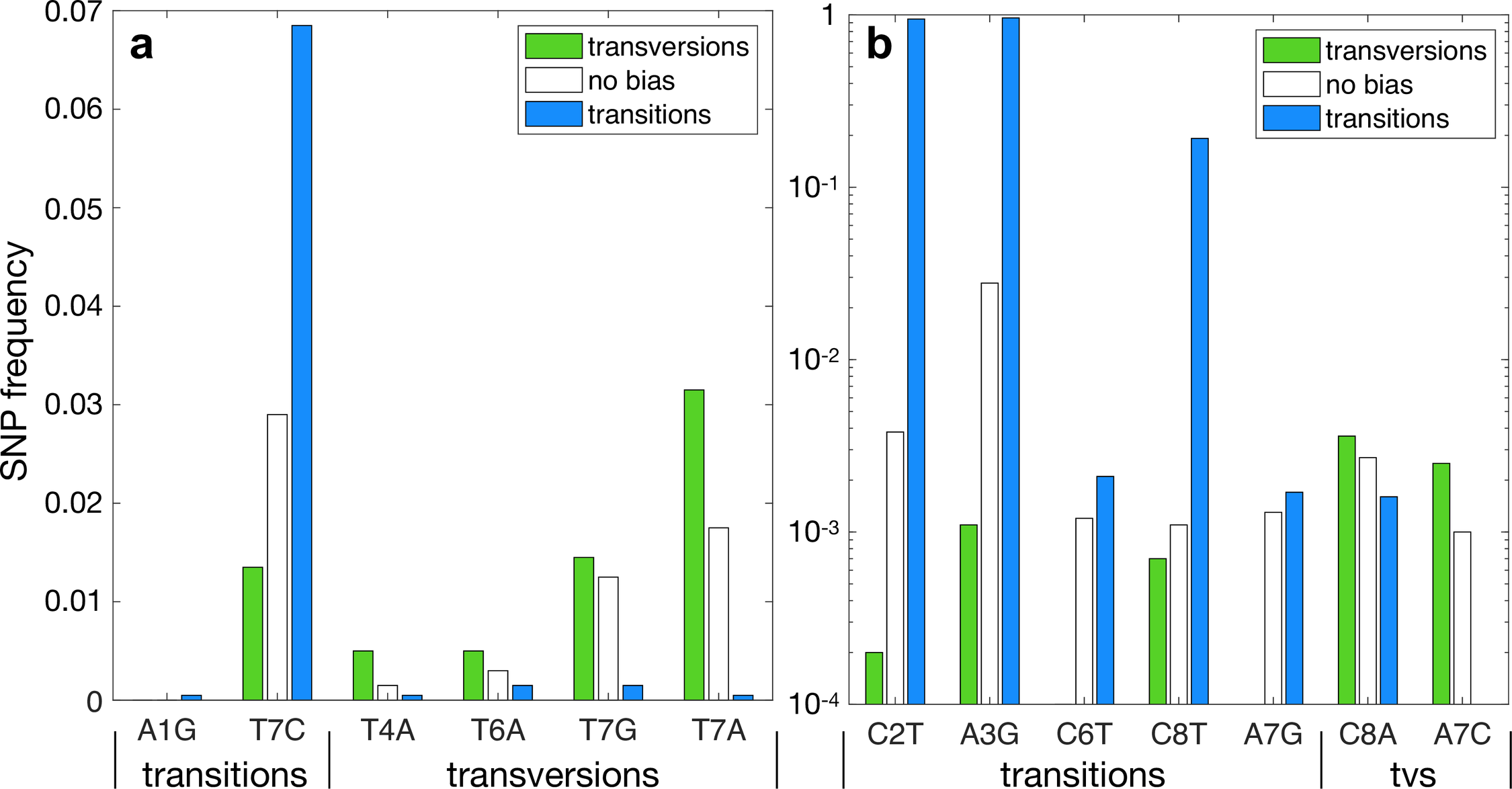

Supplement: S18 Fig — The bars correspond to polymorphisms in the population at steady state, relative to the sequence that evolved to the highest frequency in the absence of mutation bias. Bar colors correspond to three different values of the mutation bias parameter α: green for transversions (α = 0.05), white for no bias (α = 0.5) and blue for transitions (α = 0.95). The panels correspond to two Mus musculus landscapes (a) Arid5a (composition bias toward transversions) and (b) Gm397 (composition bias toward transitions). (TIF) [file pcbi.1008296.s018.tif]

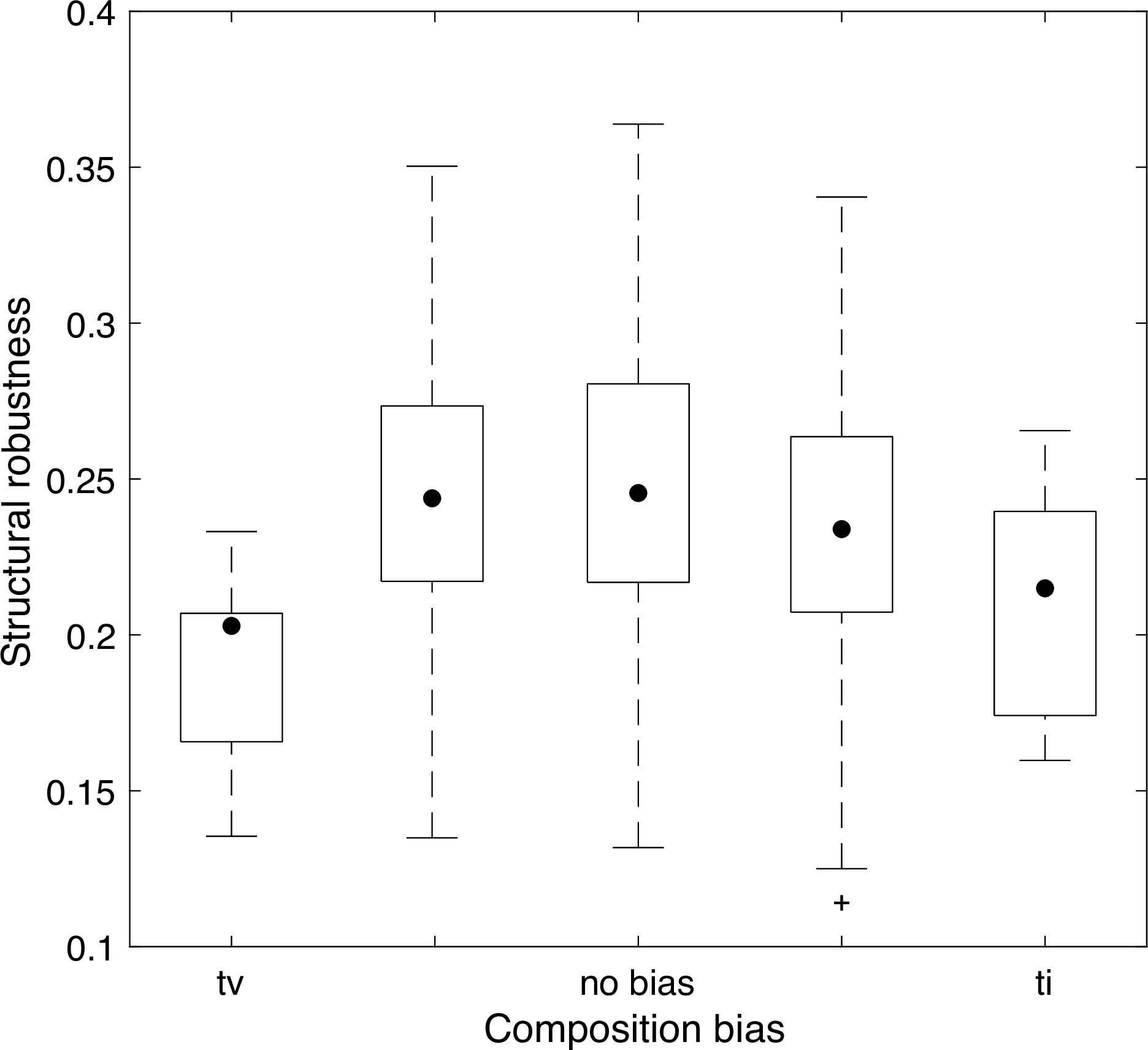

Supplement: S19 Fig — The y-axis shows the average mutational robustness of all genotypes in each landscape. The x-axis shows the composition bias. Landscapes are grouped as in Fig 3. Data pertain to all 746 landscapes. Black dots indicate medians, whiskers indicate the 25th and 75th percentiles, and cross symbols indicate outliers. (TIF) [file pcbi.1008296.s019.tif]

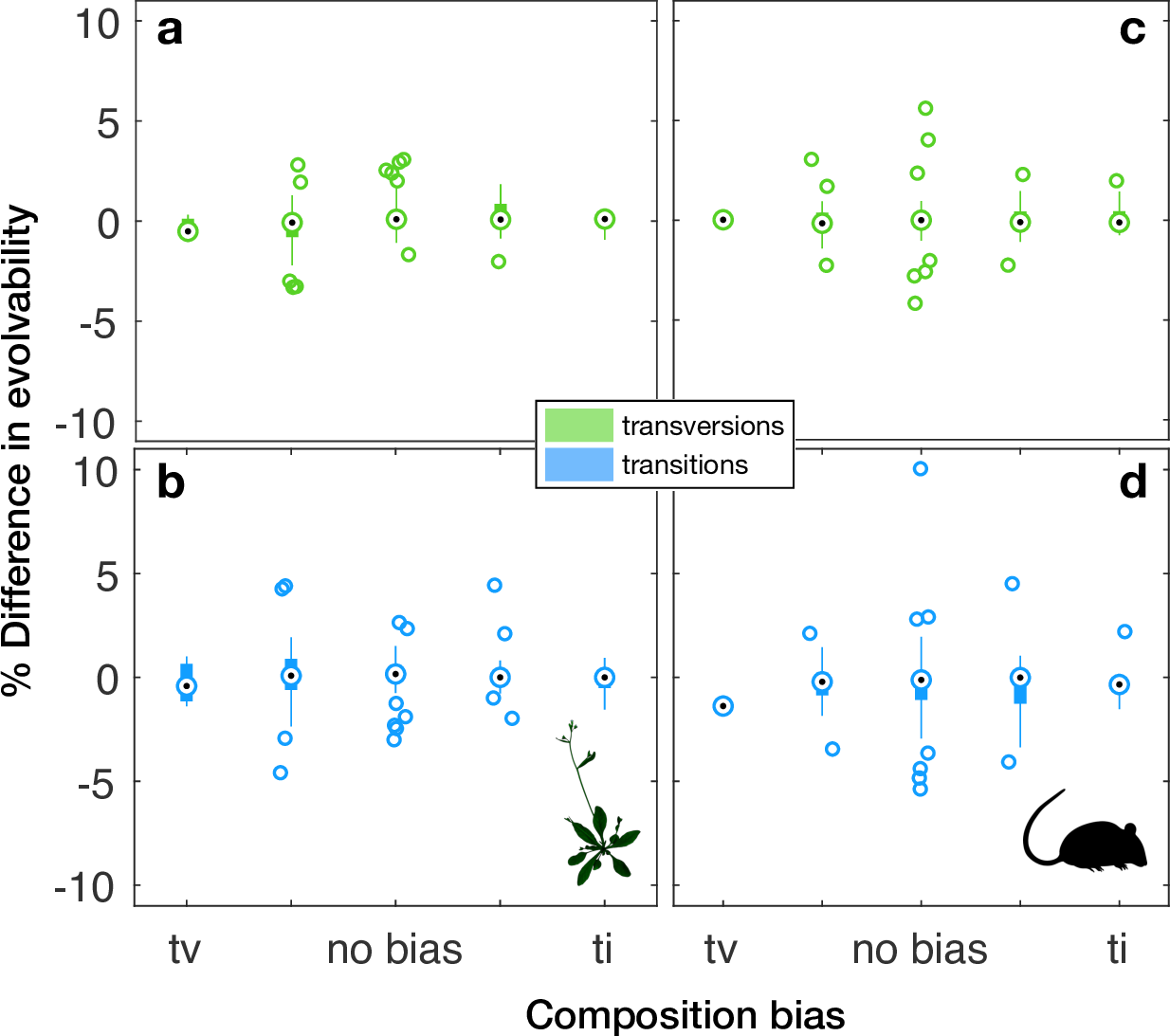

Supplement: S20 Fig — The y-axis shows the difference in evolvability, which is calculated as the difference between the evolvability of a population at steady state when there is no mutation bias and when there is a strong bias toward transversions (α = 0.05) or transitions (α = 0.95). Data pertain to 128 transcription factors from (a,b) Arabidopsis thaliana, and (c,d) 128 transcription factors from Mus musculus. Landscapes are grouped according to their composition bias as in previous figures. Parameters: N = 104, Nμ = 50. As an example, for a given transcription factor, a 10% change in evolvability under strong transition bias could mean that the one-mutant neighbors of the sequences evolved at steady state bind 11 transcription factors, whereas without mutation bias, the one-mutant neighbors of the sequences evolved at steady state bind 10 transcription factors. (TIF) [file pcbi.1008296.s020.tif]
