# Supplementary material for: Mutation bias interacts with composition bias to influence adaptive evolution
Source: PLoS Comput Biol. 2020 Sep 28;16(9):e1008296. doi: 10.1371/journal.pcbi.1008296 (PMC7571706; doi:10.1371/journal.pcbi.1008296)
Supplement: Attachment — Submitted filename: response.pdf [file pcbi.1008296.s022.pdf]

Editors' comments to the author:

Thank you very much for submitting your manuscript "Mutation bias interacts with composition bias to influence adaptive evolution" for consideration at PLOS Computational Biology.
As with all papers reviewed by the journal, your manuscript was reviewed by members of the editorial board and by two independent reviewers. In light of the reviews, below this letter, we would like to invite the resubmission of a revised version that takes into account the reviewers' comments.
We cannot make any decision about publication until we have seen the revised manuscript and your response to the reviewers' comments. Your revised manuscript is also likely to be sent to reviewers for further evaluation.
When you are ready to resubmit, please upload the following:
[1] A letter containing a detailed list of your responses to the review comments and a description of the changes you have made in the manuscript. Please note while forming your response, if your article is accepted, you may have the opportunity to make the peer review history publicly available. The record will include editor decision letters (with reviews) and your responses to reviewer comments. If eligible, we will contact you to opt in or out.
[2] Two versions of the revised manuscript: one with either highlights or tracked changes denoting where the text has been changed; the other a clean version (uploaded as the manuscript file).
Important additional instructions are given below your reviewer comments.
Please prepare and submit your revised manuscript within 60 days. If you anticipate any delay, please let us know the expected resubmission date by replying to this email. Please note that revised manuscripts received after the 60-day due date may require evaluation and peer review similar to newly submitted manuscripts.
Thank you again for your submission. We hope that our editorial process has been constructive so far, and we welcome your feedback at any time. Please don't hesitate to contact us if you have any questions or comments.

Thank you for inviting a revised submission of our manuscript.

Reviewers' comments:

Reviewer #1:

In this submission, entitled "Mutation bias interacts with composition bias to influence adaptive evolution", Cano and Payne use protein-binding array data to investigate the interplay between the mutational bias and the composition of accessible sequences along the protein-binding landscape of transcription factor binding sites. The authors show that these two properties have implications for the probability that a population will ascend to the peak of this landscape (assuming that binding affinity is equated with fitness), the amount of diversity in the population, and the robustness of the population to the disruption of protein binding.

Overall I found this paper to be quite interesting and well supported. I believe that a couple of additional analyses could strengthen the authors' case, and I describe these below, followed by a list of minor comments primarily focused on parts of the manuscript that could be edited for clarity.

Thank you for the detailed review of our work and for the positive remarks. We address your major comments below.

First, as the authors point out in the Discussion, binding affinity may not be equated with fitness (this should also be mentioned in the Introduction). I suggest that the authors experiment with creating a

simple binding phenotype->fitness mapping, and seeing how this affects their results. For example, for their simulations they could adopt a Guassian fitness function, select an optimal value, and assign fitness values to each individual based on that individuals difference from the fitness optimum, and a variance parameter analogous to the strength of stabilizing selection. The authors could then experiment with different parameters of this fitness function, and see to what extent their conclusions about landscape navigability, population diversity, and robustness are affected.

We followed the reviewer's suggestion to study the effects of a Gaussian fitness function on composition bias, assuming selection for low binding affinity as a useful counterpoint to the assumption of selection for high binding affinity made in the main text (Fig. R1). We found selection for low binding affinity transforms the topographies of the landscapes, changing the composition biases of their accessible mutational paths to the new global peaks. However, this does not affect the way in which mutation bias and composition bias interact to influence landscape navigability, population diversity, or robustness. For example, Fig. S10 shows that regardless of the particular topographical properties of the landscape under investigation, navigability is enhanced when mutation bias and composition bias are aligned and diminished otherwise. Thus, this central point of our manuscript is insensitive to whether we assume selection for low- or high-affinity binding sites. We therefore feel a detailed analysis of landscape topography under different parametrizations of a Gaussian fitness function is outside the scope of our work, and better suited for future research. Indeed, our lab has just been awarded a grant to carry out exactly this analysis.

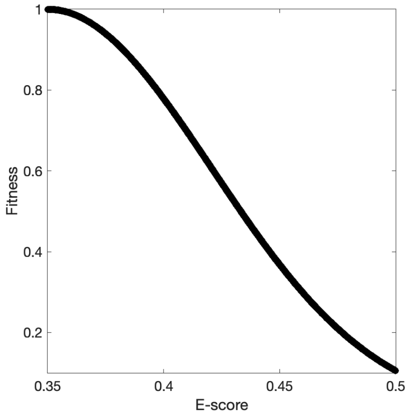


**Fig. R1. Gaussian fitness function favouring low-affinity binding sites.** Fitness is plotted as a function of binding affinity (E-score) using the Gaussian function $\exp\left(-\left((E - E_{\mathrm{opt}})/\sigma\right)^2\right)$, where E is the E-score of a binding site, $E_{\mathrm{opt}}$ is the optimal E-score, and σ is the variance parameter. Here, $E_{\mathrm{opt}} = 0.35$ (the lowest E-score in our landscapes) and σ = 0.1.

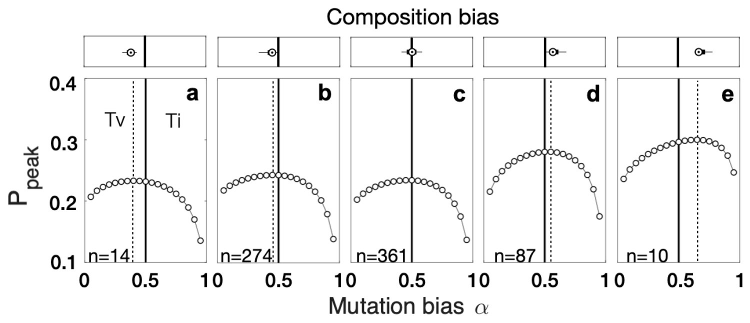

**Fig. S10. Mutation bias interacts with composition bias to influence landscape navigability, regardless of whether selection favors low or high affinity binding sites.** The probability of reaching the global $P_{peak}$ is shown for 19 different values of the mutation bias parameter. The solid vertical lines indicate no bias in mutation supply ($\alpha$=0.5) and the dashed vertical lines indicate the value of $\alpha$ that maximizes $P_{peak}$. Landscapes are grouped based on their composition bias and the distribution of composition bias per panel is shown on top of each panel. The number of landscapes per panel is indicated is the bottom left corner. Fitness is a function of binding affinity (E-score) using the Gaussian function $exp\left(-\left((E - E_{\text{opt}})/\sigma\right)^2\right)$, where E is the E-score of a binding site, $E_{\text{opt}}$ is the optimal E-score, and σ is the variance parameter. Here, $E_{\text{opt}} = 0.35$ (the lowest E-score in our landscapes) and σ = 0.1.

In response to this comment, we have added the above figure to the supplement (Fig. S10) and noted that (lines 189-192):

"*In addition, our finding that navigability will be enhanced when mutation bias and composition bias are aligned and diminished otherwise is insensitive to whether selection acts to increase or decrease binding affinity. This is important because low-affinity binding sites often drive gene expression, for example in developmental enhancers [44; 45].*"

We have also followed the reviewer's suggestion to mention early in the manuscript that selection does not always increase binding affinity (line 98):

"*…under the assumption of selection for increased binding affinity (although selection does not always act to increase binding affinity [44; 45]).*"

Second, if I understand correctly, the authors define an accessible step along the binding landscape as one that improves binding affinity above some threshold. However, this seems overly conservative to me, as all regions of the landscape that do not substantially decrease fitness should be accessible--not every mutation along a path need be beneficial. Apologies if I misunderstood this, but otherwise I suggest that the authors repeat their experiments with a second definition of accessibility: any step that doesn't decrease binding affinity by at least some threshold (perhaps the same delta used by the authors) is accessible.

We agree that defining accessible paths as those that monotonically increase binding affinity each step above some threshold is conservative. However, this definition only affects the quantification of the composition bias in the accessible paths, not the evolutionary dynamics of our simulations. For instance, in the monomorphic regime, a mutation must cause a substantial increase in binding affinity to sweep to fixation, regardless of how we define accessible mutational paths.

To understand how the definition of an accessible mutational path influences composition bias, we followed the reviewer's suggestion to consider an alternative definition, which includes steps that do not decrease binding affinity beyond some threshold (δ – our noise parameter). Fig. S6 shows that this alternative definition of accessibility results in composition biases that are highly similar to those observed under our more conservative definition. More importantly, Fig. S7 shows that composition bias measured using our more conservative definition has a slightly better correlation with the mutation bias parameter that maximizes $P_{peak}$ ($\alpha_{optimal}$). This suggests that our more conservative definition is more informative of evolutionary dynamics, justifying its place in the main text.

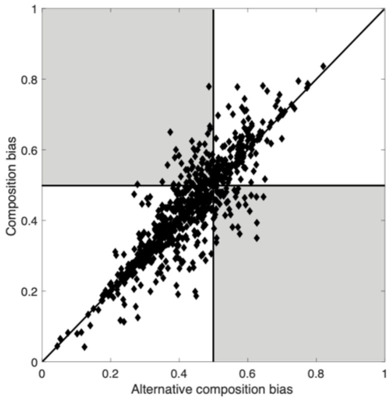


**Fig. S6. Quantifying composition bias using an alternative accessibility criteria results in very similar composition biases.** Data pertain to all 746 landscapes, Pearson's correlation coefficient ($\rho$=0.8541, p<10−12). The shaded gray regions highlight the 77 landscapes that switch from exhibiting a composition bias toward transversions to a composition bias toward transitions (or vice versa)**.**

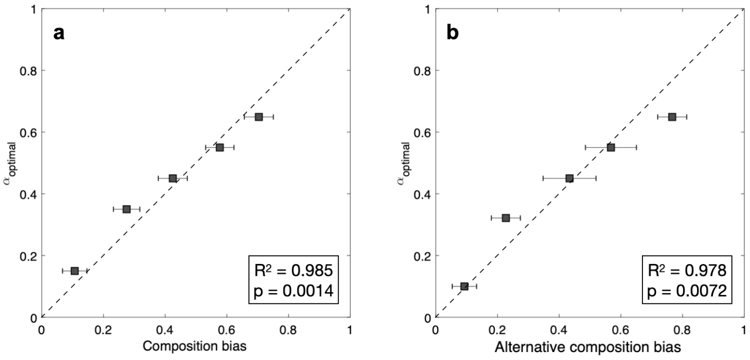


**Fig. S7. The mutation bias that maximizes $P_{peak}$ correlates strongly with composition bias, measured using two definitions of accessible mutational paths.** In **(a)**, each step in an accessible mutational path increases binding affinity by at least $\delta$. In **(b)**, each step in an accessible mutational path does not decrease binding affinity more than $\delta$. Data pertain to all 746 landscapes, each of which has its own noise threshold $\delta$.

In response to this comment, we have added the above two figures to the supplement (Figs. S6 and S7) and the following to the main text (lines 156-160):

"*In addition, we tested the sensitivity of our results to an alternative, less conservative definition of an accessible mutational path, which included any mutational steps that did not decrease binding affinity beyond some threshold ($\delta$ – our noise parameter). Fig.S6 shows that this alternative definition of accessibility results in composition biases that are highly similar to those observed under our more conservative definition, which we therefore use for the rest of our analyses, unless otherwise noted.*"

And (lines 178-179):

"*Fig. S7 shows the correlation between the mutation bias that maximizes $P_{peak}$ and composition bias, measured using two different definitions of accessible mutational paths.*"

Finally, although I enjoyed the authors' analysis of the impact of mutation/composition bias on diversity, I think it would be helpful to include a couple of additional summaries. The authors could include nucleotide diversity (pi), which would paint a very similar picture to Shannon's diversity index

but may be more familiar to some readers. I also would like to see them include allele frequency spectra, to give readers a picture of how mutation and composition bias may together influence the frequency of mutations in the population.

Good idea. We have added an analysis of nucleotide diversity in Fig S17, which closely resembles our observations with Shannon's diversity index, as you anticipated. We have also added two representative allele frequency spectra in Fig. S18, which illustrate how mutation bias and composition bias influence the frequency of mutations in a population. We have added the following text to explain these new figures (lines 279-290):

*"Calculating genetic diversity using nucleotide diversity $\pi$ results in similar patterns (FigS17). Specifically, diversity increases when mutation bias aligns with composition bias and decreases otherwise. To further illustrate how mutation bias and composition bias interact to influence population diversity, we show in FigS18 the allele frequency spectra of populations evolved on two different landscapes, which we chose as illustrative examples because of their strong composition bias toward transversions (FigS18a) and towards transitions (FigS18b). As expected, the allele frequency spectra reflect the sequence bias of the mutation process, with more transition polymorphisms present when mutation is biased toward transitions and more transversion polymorphisms present when mutation is biased toward transversions. These examples also illustrate two different ways in which the interaction between mutation bias and composition bias influence the frequency of mutations in the population. In FigS18a, polymorphisms are present, but they are rare, and the sequence that evolves to the highest frequency is the same as in the absence of mutation bias. In contrast, in FigS18b, some transition polymorphisms come to dominate the population, such that the sequence that evolves to the highest frequency is not the same as in the absence of mutation bias."*

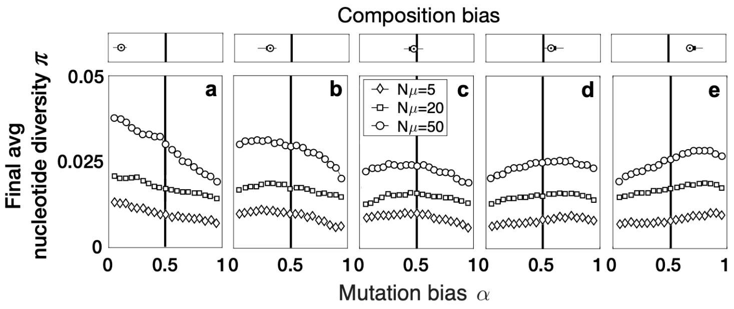


**Fig. S17. Mutation bias interacts with composition bias to influence the evolution of nucleotide diversity $\pi$. (a-e)** The final average nucleotide diversity of evolved populations at steady state is shown for 19 different values of the mutation bias parameter $\alpha$, and for each of three different values of mutation supply $N\mu$ (see legend). The solid vertical lines indicate no bias in mutation supply ($\alpha$= 0.5). Landscapes are grouped based on their composition bias and the distribution of composition bias per panel is shown on top of each panel, as in Fig. 3.

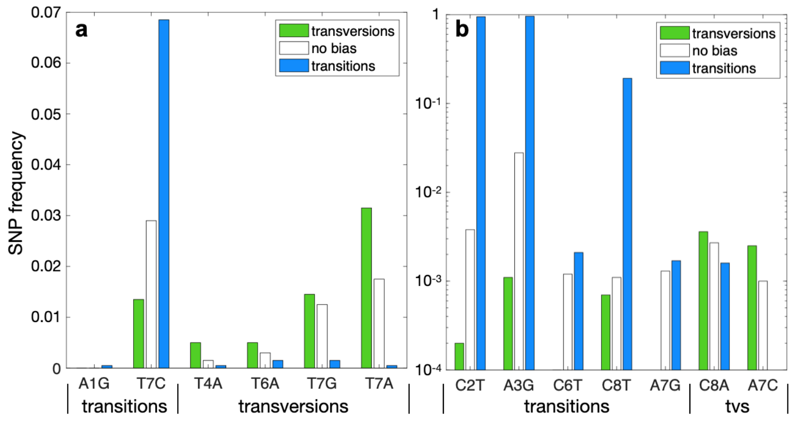


**Fig. S18. Mutation bias and composition bias interact to influence allele frequency spectra.** The bars correspond to polymorphisms in the population at steady state, relative to the sequence that evolved to the highest frequency in the absence of mutation bias. Bar colors correspond to three different values of the mutation bias parameter $\alpha$: green for transversions ($\alpha$=0.05), white for no bias ($\alpha$=0.5) and blue for transitions ($\alpha$=0.95). The panels correspond to two *Mus musculus* landscapes **(a)** Arid5a (composition bias toward transversions) and **(b)** Gm397 (composition bias toward transitions).

Minor comments:

In the intro, I would try to define composition bias more explicitly (i.e. the composition of sequences within the network/landscape, or all accessible sequences in the network in some cases).

We have edited our definition in the abstract (lines 14-15) and introduction (line 91) to read "Such composition bias – an enrichment of a particular type of mutation relative to a null expectation, throughout an entire landscape or along particular mutational paths – likely interacts with composition bias…"

Regarding path entropy: as far as I can tell path entropy is always zero or one in Figs 4a-b. Could the authors please explain this?

Figs. 4a-b do not show the distributions of path entropy, but rather the distributions of the mutation bias parameter α that minimizes path entropy. It is almost always α = 0.05 or α = 0.95 that minimizes path entropy – the two most extreme values of mutation bias considered in our study. The reason is that "the misalignment between mutation bias and composition bias usually leads to the minimization of path entropy, thus increasing predictability" as we state on lines 209-210.

Top of page 7: I find it interesting that path entropy is decreased more by transversion bias than transition bias, given that in the absence of composition bias, transition mutation bias represents a greater constraint of available paths, as argued by the authors a few sentences above. Moreover, there is a transversion composition bias on average in accessible paths, which should again result in transition bias having a stronger impact on path entropy, right? Or perhaps I am misunderstanding. In either case, could the authors please explain/discuss in the text?

The reviewer's intuition is correct. Mutation bias towards transitions is a greater evolutionary constraint in landscapes without composition bias, because there are fewer transition mutations than transversion mutations. By reporting extreme values, specifically that "path entropy decreased by **up to** 29-fold and 18-fold in the most extreme mutation biases toward transversions and transitions, respectively", we gave the false impression that path entropy was decreased more by transversion

bias than transition bias. We now report averages instead, specifically that path entropy (lines 215-217)

"*decreased by an average of approximately 2-fold and 3-fold in the most extreme mutation biases toward transversions and transitions, respectively (Fig. 4c).*"

and clarify that (lines 207-208)

"*… a mutation bias toward transitions minimized path entropy more often than one toward transversions*"

Also regarding path entropy: the value of alpha that maximizes path entropy is positively correlated with composition bias, as expected, but this seems to be a fairly weak correlation. Do the authors have any thoughts on why this is?

We thank the reviewer for this comment, because it led us to study this correlation in more detail and to realize that the weak correlation we observe is actually what we should expect. The reason is that our measure of composition bias does not capture how mutations are distributed across accessible mutational paths, which strongly influences the value of α that maximizes path entropy. This is illustrated with a simple example in Fig. S13.

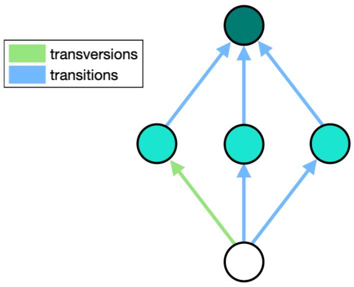


**Fig. S13. Composition bias is a weak predictor of the mutation bias α that maximizes path entropy.** Nodes represent sequences in a landscape, and directed edges represent accessible mutations between sequences. Edge colors represent mutation type and node colors represent binding affinity (darker = higher). This landscape exhibits a strong composition bias toward transitions. Path entropy is therefore minimized by a strong mutation bias toward transversions, because an evolving population will utilize only one of the three accessible mutational paths. Conversely, one might expect path entropy to be maximized by a strong mutation bias toward transitions. However, this is not the case, because an evolving population will only utilize two of the three accessible mutational paths. The mutation bias that maximizes path entropy is actually the one that makes the three first-step mutations equiprobable. In more complex scenarios, with more and longer paths that include a greater diversity of binding affinities and more heterogeneous distributions of mutation types, a single summary statistic like composition bias is unlikely to accurately predict the value of mutation bias that maximizes path entropy.

In response to this comment, we have added Figure S13 to the supplement and we have modified our text surrounding Figure 4e as follows (lines 223-228):

"*We hypothesized that the value of α that maximizes path entropy will be positively correlated with composition bias, such that path entropy will be maximized when these biases are aligned. Fig. 4e reveals this positive correlation, which is statistically significant, but weak in explanatory power (Spearman's rank correlation coefficient $\rho = 0.13$, $p < 10^{-4}$). The reason is that our measure of*

*composition bias does not capture how mutations are distributed across accessible mutational paths, which strongly influences the value of α that maximizes path entropy (Fig. S13).”*

Figure 6: I recommend simulating more replicates for each mutation bias and landscape so that the resulting plots are less noisy--might help readers see the patterns more clearly. For example, in some cases there are dips near the edges of the x-axis, and this will make it clearer whether the presence/absence of such a dip is meaningful.

We have followed the reviewer’s suggestion by increasing the number of replicate simulations per initial condition by 50% (from 10 to 15). Additionally, we now consider a revised measure of diversity, as suggested by reviewer 2. Together, these changes resulted in more smooth and clear trends.

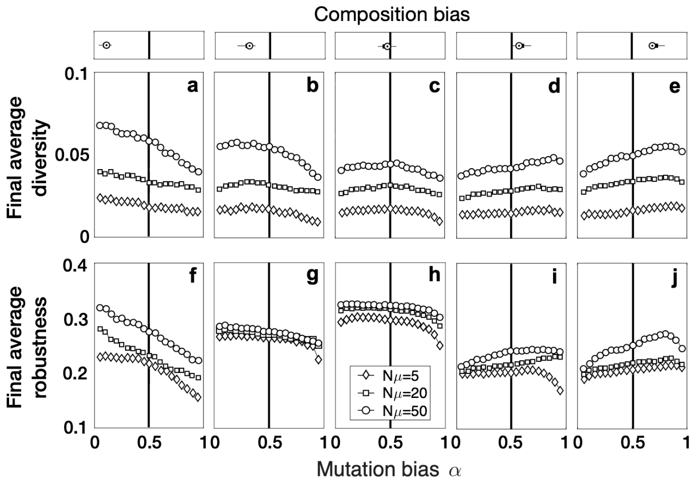


**Fig 6. Mutation bias interacts with composition bias to influence the evolution of genetic diversity and mutational robustness. (a-e)** The average genetic diversity and **(f-j)** mutational robustness of evolved populations at steady state is shown for 19 different values of the mutation bias parameter α, and for each of three different values of mutation supply Nμ (see legend). The solid vertical lines indicate no bias in mutation supply (α= 0.5). Landscapes are grouped based on their composition bias and the distribution of composition bias per panel is shown on top of each panel as in Fig. 3.

Regarding the section entitled "Mutation bias influences the evolvability of polymorphic populations": I found this section hard to understand in general, and in particular the last two sentences are kind of a word salad. I recommend a careful edit/rewrite pass for clarity.

We have added lines (325-327):

*“Said differently, the fraction of sequence space covered by these peaks is too small to facilitate mutational access to the landscapes of other transcription factors.”*

I also think that "evolvability" needs to be defined more precisely, as "the ability of mutation to bring forth new binding phenotypes" is not specific enough in this context.

We feel the precise definition is too technical to include here, but the reviewer is correct that it needs to be formally specified in the manuscript. We have therefore added a forward reference to a new paragraph of the Methods that describes this measure (lines 499-503).

*“We quantified the evolvability of a transcription factor’s binding sites as follows. First, we determined the set of binding sites that have evolved at steady state in our Wright-Fisher simulations. Then we*

*enumerated the set of DNA sequences that differ by one mutation from any of these binding sites, but are not themselves part of the focal transcription factor's landscape. Finally, we determined the fraction of transcription factors in our dataset that these one-mutant neighbors bind. This fraction was our measure of evolvability."*

Finally, in that same section, the authors state that "S11 Fig shows how a bias in mutation supply affected the evolvability of polymorphic populations for 128 transcription factors from Arabidopsis thaliana and 128 transcription factors from Mus musculus, the two species with the most transcription factors in our dataset." I don't see how Fig S11 shows this. Perhaps the authors can clarify this in the legend.

The y-axis of Fig. S20 (Fig. S11 in the previous version of the manuscript) shows the percentage change in evolvability that is induced by a strong transition or transversion bias, relative to when there is no mutation bias. To make this more concrete, we have added the following to the caption:

"*As an example, for a given transcription factor, a 10% change in evolvability under strong transition bias could mean that the one-mutant neighbors of the sequences evolved at steady state bind 11 transcription factors, whereas without mutation bias, the one-mutant neighbors of the sequences evolved at steady state bind 10 transcription factors.*"

Reviewer #2:

Recent work on evolution on genotype networks motivated by computational genotype-phenotype models has changed how we view evolution in many ways. We now know that the networked structure of genotype space can alter evolutionary outcomes in ways that we couldn't have predicted decades ago. An obvious advance in this topic is to study how mutation bias can modify this picture, by favoring some evolutionary pathways and thus affecting the chances to reach evolutionary optima. The effect of mutation bias is further compounded by composition bias, where a given landscape has an uneven proportion of nucleotides. The interaction between both biases and their effect on adaptive evolution is what the authors set to study in this paper, using a database of transcription factor-based landscapes.

The paper is well-written and relevant, and all in all I think it is more than suitable for PLoS Comp Biol. I have several minor comments that in my opinion would improve the manuscript, and also suggest further analysis and new methods in some cases: the authors are free to ignore these if they disagree with me or if what I ask is computationally expensive.

Thank you for the detailed review of our work and for the positive remarks. We address your comments point-by-point below.

Minor comments:

1) P4, L120: "For some transcription factors (~37%), the genotype network fragmented into several disconnected components. When this occurred, we only considered the largest component, which always comprised more than 100 bound sequences."

I'm wondering, when there is more than one connected component, what is the distribution of their sizes? Is it always the case that one component is super large, while the others are very small? I guess if there are components of comparable size, the authors could consider them separately and independently: in the end, they are more or less parallel evolutionary universes somehow.

It is almost always the case (97%) that the non-dominant components do not reach our size requirement of 100 sequences, which is why we do not include them in our analyses. We show this in the new Fig. S1.

We added to the manuscript the following sentence (lines 117-119):

"*We discarded non-dominant components because they usually comprised few sequences and rarely met our size requirement of 100 sequences.*"

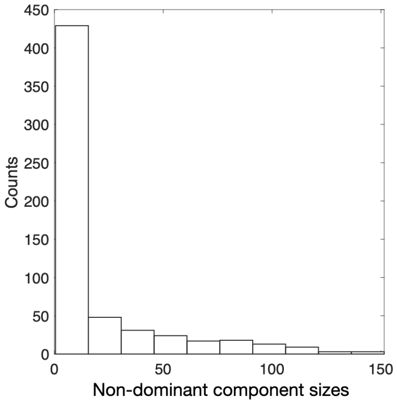


**Fig. S1 Non-dominant genotype network components usually comprise few sequences.** Histogram of non-dominant component sizes. In total, 46% are singletons and 97% are not large enough to satisfy our inclusion criterion of containing 100 sequences.

2) P5, L165: "Under these population genetic conditions, only one mutation is present in the population at any time [52], which makes the process amenable to modeling using Markov chains (Methods)."

Not exactly. The fact that there is only one mutation at a time means we can model evolutionary dynamics as a random walk in genotype space.

This is just math nitpicking, but population dynamics can be modeled using Markov chains even in the presence of several mutations at the same time. For instance, the Wright-Fisher dynamics that the authors use later in the paper is in fact a Markov chain (it's just that it is very impractical to use matrices in this case, and thus simulations are used). Markov processes appear whenever the probability of the process to jump to a given state just depends on the state the process is in now, and not on previous history. Most models of evolutionary dynamics are Markov chains.

Thank you for the clarification. We have changed the text accordingly (lines 163-165):

*"Under these population genetic conditions, only one mutation is present in the population at any time [52], which makes the process amenable to modeling as a random walk in genotype space (Methods)."*

3) In Fig3, I understand that the authors represent the average of P_peak across landscapes. I think showing the standard variation, or standard error would be informative. Is it always the case that the value of mutation bias that maximizes P_peak (for each landscape) is equal to the value of composition bias? If not, what is the average distance between these values?

In response to this comment and one made by reviewer 1, we have incorporated a new Fig. S7a that shows the value of the mutation bias parameter $\alpha$ that maximize P_peak, as a function of average composition bias.

4) In Fig4, I think showing some exemplars of alpha (x-axis) vs path entropy (y-axis) for a subset of landscapes would be very informative. How often is this function monotonous? What kind of shapes can we observe, and how are they related to composition bias? Is there any way to classify different landscapes according to this measure?

We have included a new Fig. S11 showing the relationship between path entropy and the mutation bias parameter $\alpha$ for 50 randomly chosen landscapes. Most (84%) of these curves are unimodal and few (16%) are monotonic, so we do not think that classifying them according to shape will be particularly informative.

We have incorporated to the text (lines 201-202):

*"Fig. S11 shows the relationship between path entropy and the mutation bias parameter $\alpha$ for 50 randomly chosen landscapes."*

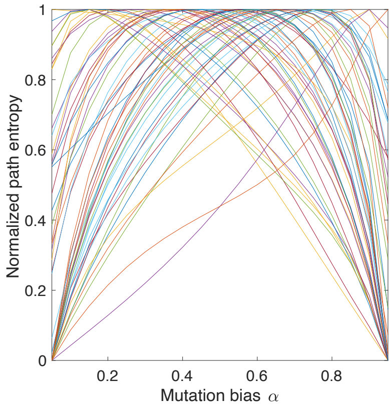


**Fig. S11. Path entropy as a function of mutation bias.** Data pertain to 50 randomly chosen landscapes for 19 different values of the mutation bias parameter $\alpha$.

5) Also in Fig4b, it looks as if alpha_min is always either 0.05 or 0.95. Is that so?

Yes. It is almost always the most extreme mutation bias that minimizes path entropy. For example, in landscapes that exhibit a composition bias towards transversions, it is usually the strongest mutation bias towards transitions that minimizes entropy, because this most severely constrains the mutational paths to the global peak.

6) In Fig5, there is (I believe) a better way to measure the overlap between steady-state populations, although I'm not sure if it's computationally feasible, it will depend on the size of the networks.

Using Wright-Fisher simulations with finite-size populations will include a stochastic effect, that the authors have measured. However, if they want to test the topological effects of mutation bias, they could simulate the evolution of infinite populations using quasispecies dynamics. In that case, the steady-state distribution of a population on a genotype network is given by the eigenvector associated to the largest eigenvalue of the evolution matrix. See, for instance, section 3 in this paper (https://royalsocietypublishing.org/doi/full/10.1098/rsob.180069). The authors could just change matrix G in equation 3.5 to have G_ij=alpha if the connection is a transition and G_ij=1-alpha if it is a

transversion, and then compute the eigenvector numerically: just multiply any initial vector by the transition matrix M, and then divide by the sum of the vectors components (so that the total sum is always one and so each component of the vector represents the probability that an infinite population is at that node), and keep doing this until the vector remains unchanged. That is the eigenvector associated with the largest eigenvalue (see also Box 1 in the same reference).

Now, this eigenvector will change with different values of alpha, and then any distance metric between vectors can be used to compare them.

This method is also valid for the results in Fig6.

We have followed the reviewer's suggestion to simulate the evolution of infinite populations using quasispecies dynamics. We have added a new section to the Methods explaining this analysis (lines 488 – 491) and a new Fig. S16 showing that in evolved infinite populations, overlap also decreases as Δ mutation bias increases.

We have added to the main text (line 262):

*"a trend that also holds for infinitely large populations (Fig. S16)."*

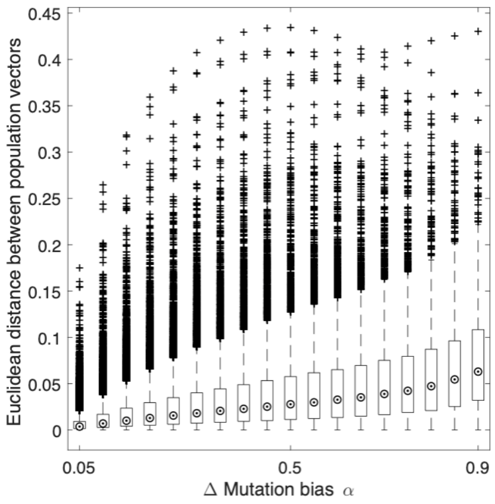


**Fig. S16. Mutation bias influences the distribution of infinite populations on a genotype-phenotype landscape.** We characterized the steady state distribution of infinite populations as the eigenvector that corresponds to the largest eigenvalue of the matrix P (Methods). For any pair of such populations, we measured their overlap as the Euclidean distance between these eigenvectors - the shorter the distance, the higher the overlap -. This panel shows this distance for pairs of populations in relation to the difference in their mutation bias parameters.

7) In Fig6, the diversity measure used is not properly normalized. H_j (as defined in equation 9) can only equal one if every possible base can be visited by the population, but this is not the case, as not all nucleotide combinations are viable. Consider the landscape {AACG, CACG, TACG}. The maximum value of H_1 is log2(3) (which is greater than 1), while H_2, H_3 and H_4 will always be 0! Properly normalizing this measure in order to compare between networks is not easy.

A different (and in my mind, more natural way) to measure diversity would be to compute Shannon's index over all genotypes. So H=\sum p_i*\log_2(p_i), where the sum ranges over all genotypes in the network. That is, p_i is the probability that the steady-state population is at a given node in the

network (and this is given naturally by the eigenvector I mentioned in my previous point). The maximum value of H here is log_2(n), where n is the size of the network, so in order to compare between networks you just need to divide H by this value.

We followed the reviewer's suggestion and recalculated the diversity index over all genotypes and then normalized it by the maximum diversity per landscape (Fig. 6).

We substituted equation 9 by:

$$H = \frac{-\sum_i p_i \log_2 p_i}{\log_2(n)}$$

where p_i is the fraction of the steady-state population that is at sequence i and n is the size of the landscape.

8) We computing mutational robustness of a steady-state population, is the average weighted with respect to the number of individuals in each genotype?

Yes.
